# Crown ethers reverse P-glycoprotein-mediated multidrug resistance in cancer cells

**DOI:** 10.1038/s41598-018-32770-y

**Published:** 2018-09-27

**Authors:** Iva Guberović, Marko Marjanović, Marija Mioč, Katja Ester, Irena Martin-Kleiner, Tatjana Šumanovac Ramljak, Kata Mlinarić-Majerski, Marijeta Kralj

**Affiliations:** 1 Division of Molecular Medicine, Ruđer Bošković Institute, Bijenička cesta 54, 10 000 Zagreb, Croatia; 2 Department of Organic Chemistry and Biochemistry, Ruđer Bošković Institute, Bijenička cesta 54, 10000 Zagreb, Croatia; 3 Present Address: Josep Carreras Leukaemia Research Institute, Campus ICO-Germans Trias i Pujol-Universitat Autònoma de Barcelona, 08916 Badalona, Spain

## Abstract

Multidrug resistance (MDR) is a widespread phenomenon exhibited by many cancers and represents a fundamental obstacle for successful cancer treatments. Tumour cells commonly achieve MDR phenotype through overexpression and/or increased activity of ABC transporters. P-glycoprotein transporter (P-gp, ABCB1) is a major cause of MDR and therefore represents a valuable target for MDR reversal. Several naturally occurring potassium ionophores (e.g. salinomycin) were shown to inhibit P-gp effectively. We have previously shown antitumour activity of a number of 18-crown-6 ether compounds that transport potassium ions across membranes. Here we present data on P-gp inhibitory activity of 16 adamantane-substituted monoaza- and diaza-18-crown-6 ether compounds, and their effect on MDR reversal in model cell lines. We show that crown ether activity depends on their lipophilicity as well as on the linker to adamantane moiety. The most active crown ethers were shown to be more effective in sensitising MDR cells to paclitaxel and adriamycin than verapamil, a well-known P-gp inhibitor. Altogether our data demonstrate a novel use of crown ethers for inhibition of P-gp and reversal of MDR phenotype.

## Introduction

Multidrug resistance (MDR) is a phenomenon that describes cross-resistance of cancer cells to a broad range of structurally diverse chemotherapeutics. Despite major advances in cancer research, MDR remains one of the main obstacles for devising successful cancer treatments. One of the main hallmarks of MDR phenotype is the overexpression of ATP-binding cassette (ABC) transporters. ABC transporters are transmembrane proteins with a wide spectrum of substrates. ABC transporters maintain the concentration of chemotherapeutics in cancer cells below cytotoxic levels. The mechanism of action relies on ATP-dependent drug efflux activity, which enables significant conformational change of the transporter to allow substrate movement across the membrane^1^.

P-glycoprotein (P-gp) belongs to the ABC transporter superfamily and is encoded by ABCB1, also known as multidrug resistance 1 (MDR1) gene. This 170 kDa transmembrane protein is mainly localized in the plasma membrane where it acts as an efflux transporter for a wide variety of structurally and chemically diverse substances. Its main function is toxin clearance, including chemotherapeutics. Therefore, the overexpression of P-gp has been a major cause of MDR in cancer and one of the main reasons for tumour therapy failure. Up to half of all human cancers have P-gp levels high enough to display MDR phenotype. Additionally, its elevated expression has been well associated with poor outcome in several cancers^1–3^. As a result, the inhibition of P-gp is regarded as one of the most promising approaches for reversing the MDR phenotype and hence, for the successful treatment of cancer. Indeed, co-administrating P-gp modulators together with anticancer drugs has been recognized as a promising strategy in the clinic for managing P-gp-mediated MDR. Despite considerable efforts, there is still no specific P-gp inhibitor that has been approved for the market^4^.

Cancer stem cell (CSC) populations are regarded as one of the most resistant cell populations within a tumour and are postulated to be the main reason for cancer relapse. CSCs resistance to chemo- and radiotherapy arises from several different mechanisms, which include increased expression of ABC drug efflux pumps (e.g. P-gp, ABCG2)^5–7^. Recently Gupta *et al*. detected salinomycin and nigericin as compounds with selective toxicity to CSCs^8^. Additionally, several studies reported that these two well-known K^+^/H^+^ ionophores show inhibitory activity towards P-gp, and/or result in the sensitization of resistant leukaemia cancer cells to different chemotherapeutics (e.g. adriamycin, docetaxel, vinblastine)^9–11^.

The main function of ionophores is their ability to transport ions across lipid membranes. This leads to disrupting cell membrane potential and ion homeostasis, which results in physiological and osmotic stress. Consequently, various natural ionophores (e.g. salinomycin, nigericin, valinomycin) are used as antibiotics, and more recently in cancer treatment^10–13^.

Our group’s research has focused on characterising the biological activity of novel crown ether compounds, as well as their chemical synthesis. Crown ethers are macrocyclic polyethers containing three to twenty oxygen atoms separated by two or more carbon atoms, which can be either substituted or unsubstituted. The most important characteristic of crown ethers is their selective binding of metal cations, anions, and neutral molecules, which qualifies them for use in a variety of applications. Crown ether complexation properties can be modified by introducing structural changes with respect to the size of the ring, substituents, or the type of donor atoms^14,15^. Due to their distinctive structure, crown ethers can transport ions across membranes and act in a similar way to natural ionophores. Ionophoric properties of crown ethers have been studied with respect to their antibacterial and antiparasitic activities, and more recently, antitumor activity^14^. Moreover, certain crown ethers have been shown to inhibit P-gp-mediated efflux of antitumor drugs, such as anthracyclines^16^.

**Figure 1:**
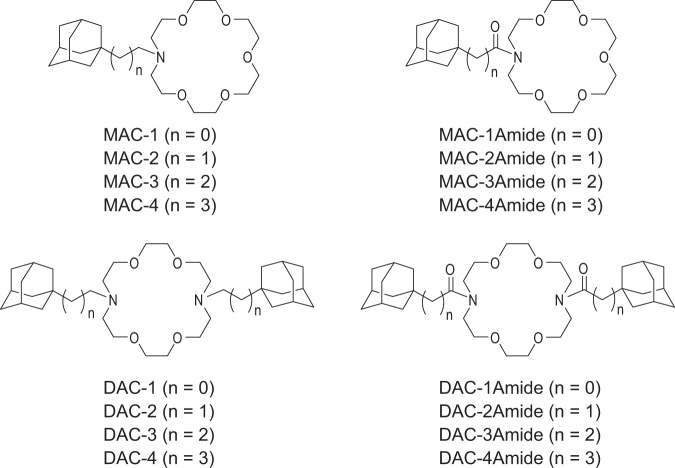
Structures of monoaza-crown ethers (MACs) and diaza-crown ethers (DACs).

We have previously developed a computational model, which enabled us to synthesise novel 18-crown-6 ether compounds. We also identified significant cytotoxicity of these novel crown ethers towards five different cancer cell types^17–19^. Based on available research, we hypothesized that 18-crown-6 ethers might show inhibitory activity towards P-gp, in addition to inhibiting tumour cell growth. As such, we supposed that they might sensitize MDR cancer cells to conventional chemotherapeutics. Therefore, we sought to elucidate mechanisms of action of 16 structurally related adamantyl-substituted monoaza- (MAC) and diaza- (DAC) crown compounds (Fig. 1), with the focus on their effect towards P-gp mediated MDR phenotype. In order to describe the biological properties of the compounds, we used an MDR cell model. We found that, in addition to cytotoxic activity towards cancer cells, a subgroup of DAC-amide compounds shows a significant inhibitory potential towards P-gp. Further, co-treating MDR cells with DAC-amide crown ethers and representative chemotherapeutics results in a pronounced reversal of the MDR phenotype. These properties were shown to be directly linked to lipophilicity and specific structural features of DAC-amide compounds.

## Results

### Synthesis of crown ethers

The synthesis of monoaza-crown ethers and diaza-crown ethers was based on *N*-alkylation of monoaza- and diaza-18-crown-6 with the corresponding adamantyl tosylates or the coupling reactions with corresponding acyl chlorides followed by reduction of the obtained crown ethers with LiAlH_4_. Detailed syntheses of MAC-2 to MAC-3Amide and DAC-1 to DAC-3Amide are shown in Supplementary information^20,21^. Syntheses of new compounds: MAC-1, MAC-1Amide, MAC-4, and MAC-4Amide, as well as DAC-4 and DAC-4Amide are described in the Supplementary information of this paper (Supplementary Figs S1 and S2).

### Evaluation of MDR phenotype in A2780/Adr cells

As a model for studying MDR related to overexpression of P-gp, we used ovarian carcinoma cell line A2780 and its adriamycin (doxorubicin) resistant counterpart A2780/Adr. Western blot analysis confirmed increased expression of P-gp in A2780/Adr cells in comparison to parental A2780 cells (Supplementary Fig. S3). In order to account for MDR phenotype, we evaluated cytotoxic effects of structurally diverse chemotherapeutics and ionophores towards A2780 and A2780/Adr. As expected, A2780/Adr cells showed cross-resistant phenotype. Accordingly, in addition to resistance towards adriamycin, they exhibited resistance to other structurally unrelated compounds, such as paclitaxel, etoposide, valinomycin, salinomycin, nigericin and verapamil (Supplementary Fig. S4). Taken together, these results confirm A2780/Adr cell line as an appropriate model for studying P-gp-mediated MDR.

### Crown ethers inhibit tumour cell viability

The effects of crown ether compounds on viability of MDR model cell lines were tested by MTT assay. Growth inhibition curves (Supplementary Fig. S5) and related IC_50_ values (Table 1.) indicate that tested crown ether compounds inhibit viability of both cell lines. Moreover, A2780/Adr cells are slightly more resistant to most crown ether compounds. DAC compounds effectively inhibited viability of both cell lines. However, DACs with an amide bond linking adamantane moiety to aza-18-crown-6 have considerably lower cytotoxicity in comparison to DACs linked by an amine bond (i.e. DAC-2Amide, -3Amide and -4Amide *vs*. DAC-2, -3 and -4). The exceptions to the “rule” are DACs with the shortest side chain (DAC-1 and −1Amide). This correlates to their affinity for alkali cations (especially K^+^) and better extraction capabilities (Supplementary Tables S1 and S2)^19,20^.

**Table 1:** *In vitro* growth inhibition of A2780 and A2780/Adr cell lines by crown-ethers.

Cmpd	IC_50_ (μM)		ALOGP	Cmpd	IC_50_ (μM)		ALOGP
	A2780	A2780/Adr			A2780	A2780/Adr	
MAC-1	24 ± 4	39 ± 32	1.96	DAC-1	21 ± 1.8	50 ± 6	4.70
MAC-1Amide	19 ± 12	58 ± 44	1.30	DAC-1Amide	2 ± 0.8	3 ± 3	3.38
MAC-2	12 ± 1	26 ± 6	2.28	DAC-2	1 ± 0.2	2 ± 0.02	5.35
MAC-2Amide	13 ± 1	20 ± 4	1.33	DAC-2Amide	5 ± 1.7	20 ± 5	3.45
MAC-3	9 ± 5	19 ± 10	2.74	DAC-3	2 ± 0.5	8 ± 2	6.25
MAC-3Amide	11 ± 1	14 ± 0.9	1.79	DAC-3Amide	18 ± 4	32 ± 7	4.36
MAC-4	3 ± 1	11 ± 0.1	3.19	DAC-4	2 ± 0.1	4 ± 0.4	7.17
MAC-4Amide	5 ± 3	15 ± 1	2.25	DAC-4Amide	18 ± 3	46 ± 15	5.28

Diaza-crown ethers’ (DACs) and monoza-crown ethers’ (MACs) IC_50_ values (mean ± s.d. of three individual experiments performed in quadruplicates) on indicated cell lines, along with the calculated ALOGP values for each structure. (IC_50_ - the concentration that causes 50% of cell viability; ALOGP was calculated according to on-line software ALOGPS2.1, http://www.vcclab.org/lab/alogps/.

On the other hand, MAC compounds inhibit cell viability to similar extent, independently of the type of link between adamantane and aza-18-crown-6 (amide *vs*. amine). Increase in MAC activity can be observed in compounds with longer side chains and higher lipophilicity, as shown in Table 1.

### P-gp drug efflux is affected by crown ethers

**Figure 2:**
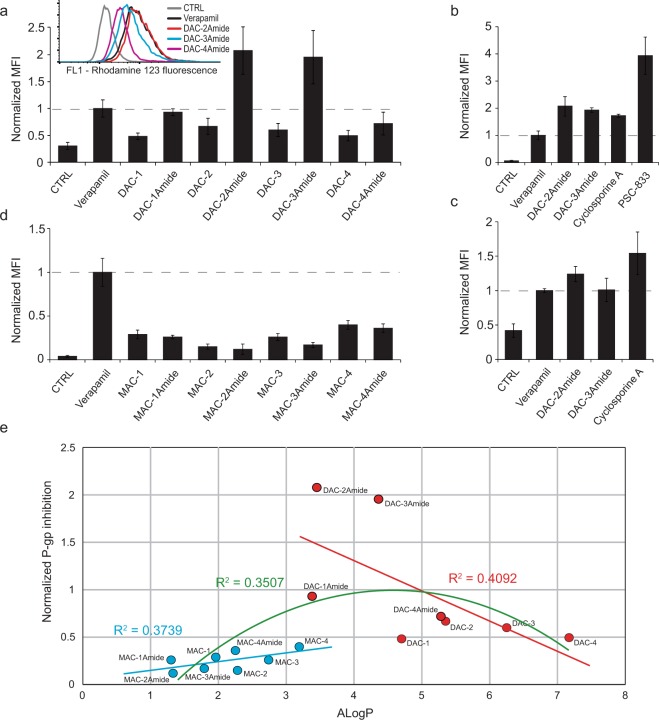
P-gp drug efflux is affected by crown ethers. Functional activity of P-gp was evaluated by Rhodamine 123 efflux assay by flow cytometry. Normalized median fluorescence intensity (MFI) of Rhodamine 123 in A2780/Adr cells after incubation with 0.5 µM Rhodamine 123 (CTRL) and treatment with verapamil and diaza-crown ethers (**a**) or monoaza-crown ethers (**d**) as indicated. Representative flow cytometry histogram of Rhodamine 123 after treatment with indicated compounds is shown in inlet. (**b**) A2780/Adr cells treated with verapamil, DAC-2Amide, DAC-3Amide, cyclosporine A and PSC-833. (**c**) Normalized MFI of Rhodamine 123 in MDR1-MDCKII cells after treatment with verapamil, DAC-2Amide, DAC-3Amide and cyclosporine A as indicated. All data are shown as mean ± s.d. of 3 individual experiments and normalized to verapamil. (**e**) P-gp inhibition for MAC and DAC compounds in Rhodamine 123 efflux assay (from **a** and **b**) was correlated to AlogP values (from Table 1). Linear regression fit for MACs (blue line) and DACs (red line) and polynomial regression fit for all compounds (order 2, green line). R^2^ values are indicated above trend lines in complementary colour.

To test the inhibitory potential of crown ether compounds towards P-gp we used 10 µM concentration of all compounds. Selected concentration enabled testing without any toxic effect on the cells, and is in accordance with previously published data for verapamil (first generation P-gp inhibitor)^3,4^. All diaza-crown derivatives decreased efflux of a fluorescent P-gp substrate rhodamine 123 (Rho123, Fig. 2a). However, only verapamil and compounds DAC-2Amide and -3Amide caused a statistically significant retention of Rho123 (p < 0.05 for verapamil, and p < 0.001 for DAC-2Amide and -3Amide). Compounds DAC-2Amide and -3Amide demonstrated inhibitory activity that was more prominent than verapamil (p < 0.001 and p < 0.01 respectively). None of the other compounds induced statistically significant Rho123 efflux inhibition compared to verapamil. Moreover, DAC-2Amide was comparable, but slightly less active than cyclosporine A (CsA), and much less potent than PSC-833 (Fig. 2b,c). We additionally tested DACs’ inhibitory effect towards P-gp in an alternative test, calcein accumulation assay. Similarly, all diaza-crown compounds led to increased accumulation of calcein within resistant cells (Supplementary Fig. S6). Compounds DAC-2Amide, -3Amide and -1Amide showed the most potent P-gp inhibition, comparable to that of verapamil.

To confirm obtained results, we assessed P-gp mediated efflux of Rho123 in another MDR cell model – MDCKII-MDR1 cells with P-gp overexpression (Supplementary Fig. S3). DAC-2Amide and -3Amide inhibited Rho123 efflux from MDCKII-MDR1 cells to a similar extent (Fig. 2c). The inhibition was similar to verapamil and slightly lower than CsA.

On the other hand, MACs showed lower inhibitory potential towards P-gp than DACs, with only MAC-4 showing statistically significant decrease of Rho123 efflux in A2780/Adr cells (p < 0.05), compared to control cells (Fig. 2d). However, all compounds were significantly less active than verapamil (p < 0.001).

Moreover, MACs’ activities could be correlated to their AlogP values (calculated according to the on-line software ALOGPS2.1^22^, Fig. 2e). Correlation analysis of P-gp inhibitory effect and AlogP values showed linear regression for MACs (R^2^ = 0.3739) and DACs (R^2^ = 0.4092) with optimal AlogP value for the best P-gp inhibition around 4. As the slopes of linear trend lines fitted for MAC and DAC compounds are opposite, the best regression fit for all compounds together (MACs and DACs) is polynomial with order 2 (R^2^ = 0.3507; Fig. 2e).

### Crown ethers sensitize P-gp overexpressing cells towards paclitaxel and adriamycin

**Figure 3:**
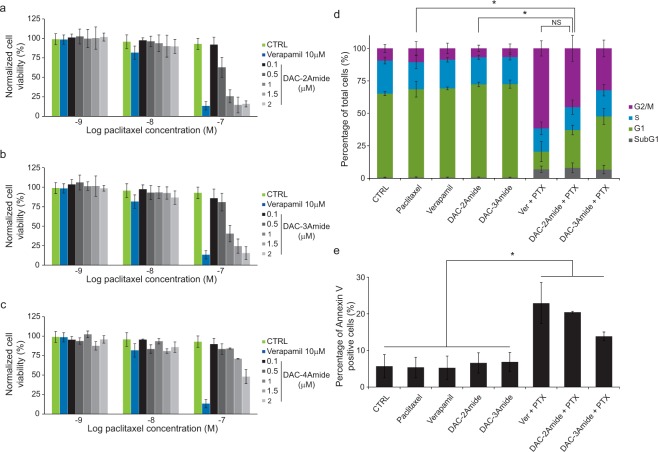
Sensitization of resistant A2780/Adr cells to paclitaxel. A2780/Adr cell line was treated with increasing concentrations of paclitaxel (PTX) alone or in combination with verapamil or DAC-2Amide (**a**), DAC-3Amide (**b**) or DAC-4Amide (**c**) at concentrations as indicated. The cell viability was evaluated by MTT assay after 72 h of incubation, and the percentage of viable cells was calculated. Cell cycle (**d**) and apoptosis (**e**) assessment: A2780/Adr cells were cultured without treatment (CTRL), treated with compounds alone as indicated (0.1 µM paclitaxel, PTX; 10 µM verapamil, Ver; 1 µM DAC-2Amide and -3Amide), or in combination with paclitaxel as indicated for 48 h (SubG1 – cell with less than 2n DNA content; G1, S and G2/M phases of cell cycle). Each bar represents a mean value ± s.d. of at least three individual experiments performed in quadruplicates (**a**–**c**) or n = 3 individual experiments (**d**,**e**). One-way ANOVA with Tukey’s post-hoc test was used for statistical analysis (NS – non-significant; *p < 0.05).

Based on the obtained results, we decided to test if crown ethers can sensitize A2780/Adr cells to paclitaxel (PTX) by treating them with non-toxic concentrations of the most active compounds (DAC-2Amide, -3Amide and -4Amide; 0.1–2 µM) (Supplementary Fig. S7). Interestingly, compounds DAC-2Amide and -3Amide lead to pronounced sensitization of A2780/Adr cells to PTX, comparable to verapamil (Fig. 3a,b, and Table S3). Compound DAC-4Amide sensitized the resistant cells towards PTX, but to a lesser extent (Fig. 3c), while the compound DAC-2 did not lead to cell sensitization to PTX (data not shown).

Furthermore, to demonstrate that the sensitization is not specific for PTX, we examined sensitization of resistant cells to adriamycin. Similarly to PTX, compound DAC-2Amide was the most potent and led to better cell sensitization towards adriamycin than verapamil (Supplementary Fig. S8 and Table S3).

In contrast, the effect of combination treatment with PTX and DAC-2Amide, -3Amide or -4Amide of non-resistant A2780 cells (Supplementary Fig. S9), can be ascribed to the slight antiproliferative effect of DACs themselves at concentrations used (Supplementary Fig. S10).

To validate that the sensitization of A2780/Adr by crown ethers is a consequence of P-gp inhibition and consequent effect of PTX, we analysed the effect on cell cycle perturbations in resistant cells. PTX stabilizes microtubules and inhibits their de-polymerisation leading to mitotic arrest, inhibition of proliferation, and eventually cell death in non-resistant cells^23^. Results demonstrate that the treatment with PTX, verapamil, or tested compounds alone did not affect the cell cycle in resistant A2780/Adr cells. On the contrary, the co-treatment with PTX and DAC-2Amide or DAC-3Amide, induced a notable G2/M arrest, along with the increase of subG1 population, indicative of cell death (Fig. 3d and Supplementary Fig. S11). Although the combination of PTX and verapamil induced the most prominent G2/M arrest, it is comparable to combination treatment with DAC-2Amide or -3Amide, which were used in lower concentration.

To confirm that the increased subG1 population of cells (Fig. 3d) is apoptotic, we analysed cells treated with combination of PTX and DAC-2Amide or -3Amide using the Annexin V assay. In comparison to compounds alone, co-treatment of cells with PTX and verapamil or crown ethers significantly increased the number of apoptotic cells (Annexin V positive cells; Fig 3e and S12). Both cell cycle arrest results and increase of apoptotic cells, support the finding that DAC-2Amide and -3Amide sensitize cells to PTX in resistant A2780/Adr cells.

### Crown ethers do not induce UIC2 shift

**Figure 4:**
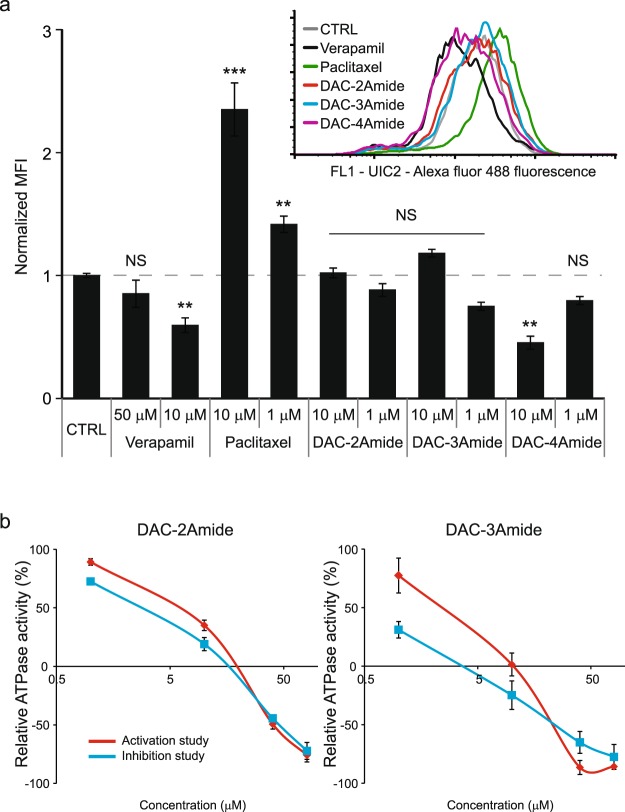
Crown ethers are not P-gp substrates. UIC2 shift assay (**a**) was used to evaluate the impact of verapamil, paclitaxel, and DAC-2Amide, -3Amide and -4Amide on P-gp conformation. Normalized median fluorescence intensity (MFI) upon treatment with compounds at concentrations as depicted. Inlet shows representative flow cytometry histograms of A2780/Adr cell line treated with 10 µM concentration of compounds as indicated. Each bar represents a mean value ± s.d. of three individual experiments. One-way ANOVA with Dunnett’s post-hoc test was used for statistical analysis (NS – non-significant; *p < 0.05, **p < 0.01, ***p < 0.001). (**b**) Relative ATPase activity after treatment of P-gp enriched vesicles with compounds DAC-2Amide and DAC-3Amide. Results shown as mean ± s.d. are representative of 2 independent experiments performed in duplicates.

Upon observing that crown ethers inhibit P-gp activity, we decided to study their mechanism of action. To test if they might act as P-gp substrates and exert their inhibitory activity via competitive inhibition, we used UIC2 shift assay. Substrates and competitive inhibitors induce a conformational change in P-gp. This is recognized by UIC2, a conformation-sensitive monoclonal antibody specific for P-gp external epitope. Cells treated with known P-gp substrate (e.g. paclitaxel) show increased binding of UIC2 (shift to the right)^24–26^. We confirmed the increased reactivity of UIC2 antibody with P-gp upon the addition of PTX, while compounds DAC-2Amide and -3Amide did not lead to the shift in comparison with non-treated cells (Fig. 4a). Surprisingly, the treatment of cells with 10 µM verapamil or DAC-4Amide, induced a negative shift (decrease of UIC2 binding) similarly to the previously described allosteric inhibitor cis-(Z)-flupentixol^25^.

### Crown ethers modulate ATPase activity

The nature of interaction between compound and transporter can be assessed by an *in vitro* P-gp-ATPase assay. This assay measures two different modes: ATPase activation and ATPase inhibition^27^. Both DAC-2Amide and -3Amide inhibited ATPase activity in a concentration dependent manner (Fig. 4b, inhibition study). Interestingly, both compounds also activated ATPase at 1 µM concentration in the activation study. However, we observed a decrease of ATPase activity with increasing concentrations of compound, which is contrary to what would be expected for ATPase substrate. Besides, with increasing concentrations of the compounds, ATPase activity diminished even below its basal activity (DAC-2Amide and -3Amide at 40 and 80 µM). We noticed that the treatment of cells with very high concentrations (up to 100 µM) of crown ethers almost immediately negatively influenced the viability of cells (data not shown). Overall, the results obtained in UIC2 shift and ATPase assays indicate that crown ethers are probably not P-gp substrates.

### Crown ethers do not affect P-gp expression, but modulate intracellular signalling networks

In addition to efflux inhibition, an effective way of reversing MDR phenotype can be achieved through manipulation of P-gp expression. Since our results did not lead to a straightforward conclusion about inhibitory mechanism of tested crown ethers, we analysed if they might affect P-gp expression. PI3K/Akt (AKT1) and MEK/ERK (MAPK2 and MAPK1, respectively) signalling are known to be involved in the modulation of P-gp expression^28,29^. Therefore, we investigated if crown ether compounds exert their activity through these signalling pathways.

**Figure 5:**
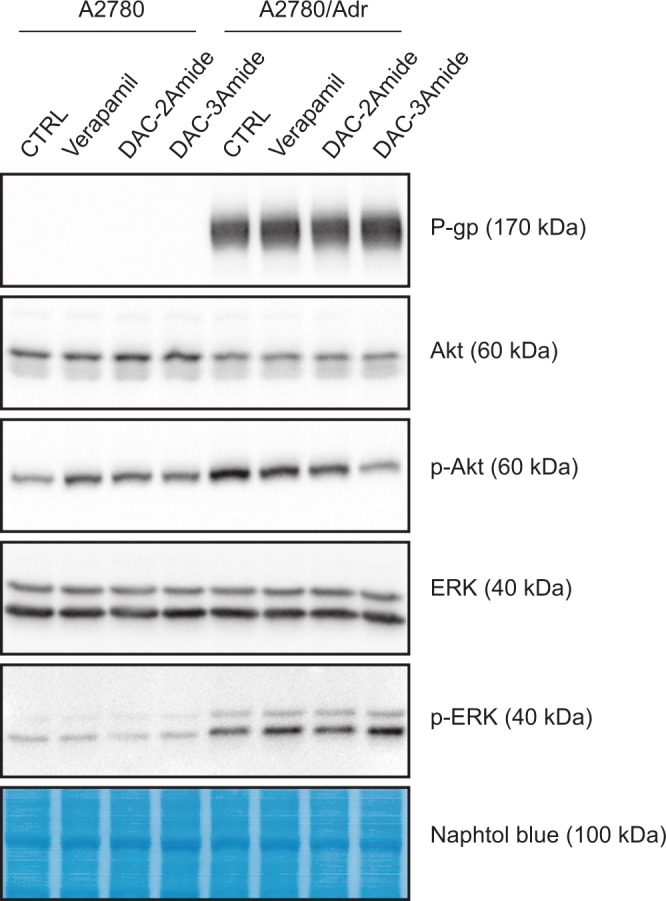
Cellular signalling proteins affected by crown ethers. A2780 and A2780/Adr cells were cultured for 72 h without treatment (CTRL) or treated with verapamil (10 μM,) or DAC-2Amide and DAC-3Amide (2 μM). Western blot was performed on total cell lysates with antibodies against indicated proteins. Naphtol blue staining was used as a loading control (bottom panel). Full length blot images are presented in Supplementary Fig. S14.

We noticed that DAC-2Amide and -3Amide did not alter P-gp expression in any of the cell lines after 72 hours treatment (Fig. 5), nor after a prolonged treatment of 10 days (data not shown). Interestingly, verapamil did not change P-gp expression in resistant cells under the same conditions (Fig. 5). This is contrary to several studies that demonstrated a decrease in P-gp expression after the treatment with verapamil^30,31^.

Furthermore, a clear increase in the level of p-Akt and p-ERK was observed in the resistant A2780/Adr compared to the parental A2780 cell line, which correlated well with P-gp expression. Interestingly, resistant cells show increased p-Akt levels, in spite of lower levels of total Akt in comparisson to parental cell line. Similar correlation of activated Akt was already described in other P-gp overexpressed cell lines^28^. The treatment with DAC-2Amide and -3Amide reduced Akt phosphorylation in A2780/Adr cells compared to non-treated cells, with more pronounced effect elicited with DAC-3Amide. On the other hand, parental and resistant cell lines did not express different levels of ERK, but resistant cells showed elevated levels of p-ERK (Fig. 5). Still, no clear difference could be noticed in total ERK nor its phosphorylation after treatment with crown ethers or verapamil. Therefore, although crown ethers modulate intracellular signalling networks, they do not affect P-gp expression.

### Crown ethers do not affect the localization of P-gp protein

**Figure 6:**
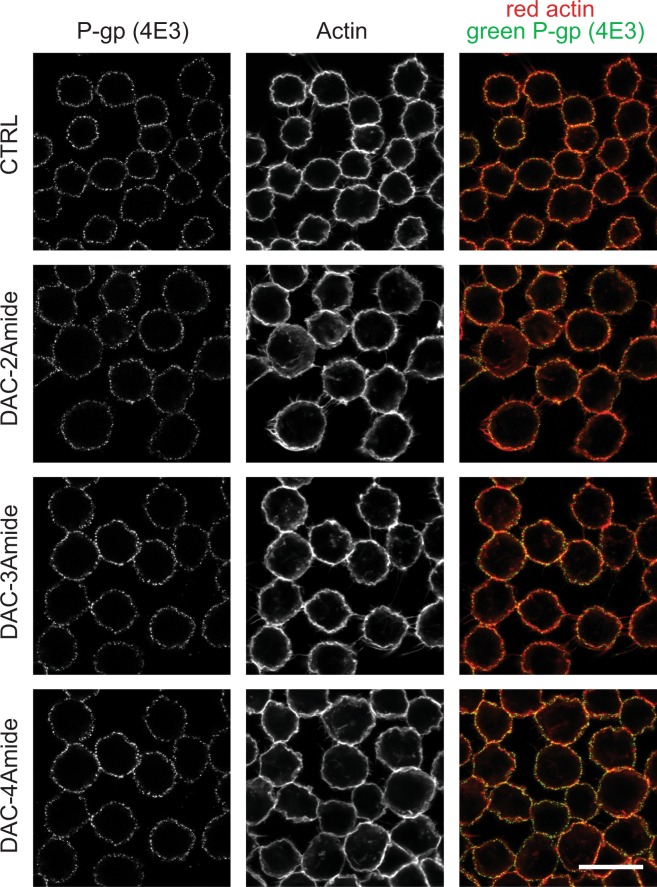
P-gp expression at the plasma-membrane is not affected by crown ethers. A2780/Adr cells were cultured without treatment (CTRL) or treated with compounds DAC-2Amide, -3Amide or -4Amide (2 μM) for 72 h. Immunofluorescent staining with phalloidin (actin, red) and anti-P-gp antibody (clone 4E3, green). One optical section shown (middle plane; scale bar = 20 μm).

P-gp-mediated drug efflux is optimal when the transporter is localized at the plasma membrane^32^. Since we did not detect difference of total P-gp level after long-term exposure to crown ethers (Fig. 5), we were interested to see if there was an effect on its plasma membrane localization or trafficking. Disrupting the intracellular trafficking of P-gp may provide a potential pathway for overcoming MDR in chemotherapy, e.g. by inhibiting the interaction of P-gp and actin^33^. Here, we show by immunofluorescence that treatment of A2780/Adr cells with low-doses of DAC-2Amide, -3Amide and -4Amide for 72 hours did not diminish the expression of P-gp at the plasma membrane (Fig. 6). Quantification of P-gp expression by flow cytometry displayed slight increase that was not statistically significant (Supplementary Fig. S13A; p > 0.05). Also, no difference in its expression was observed when immunofluorescence was monitored with P-gp antibody that recognizes the inner epitope of the transporter (Supplementary Fig. S13B). However, we noticed that treatment with DAC-2Amide results in a minor loss of Rho123 efflux efficiency (supplementary Fig. S13C; p > 0.05). Together, these results reveal that prolonged exposure to crown ethers does not affect the localization of P-gp at the cell membrane.

## Discussion

Multidrug resistance (MDR) is considered a major obstacle for successful chemotherapy and is often a result of increased expression of ATP-binding cassette (ABC) transporters. P-glycoprotein (P-gp, ABCB1) is one of the most significant causes of MDR and thus represents an attractive target for intervention^1–3,34^. Nevertheless, P-gp inhibitors are still unavailable on the market due to the lack of adequate clinical efficacy, various pharmacokinetic interactions and safety concerns. Continuous efforts to describe the structure and function of this protein in greater detail should help improve targeting P-gp-mediated MDR in the future. Therefore, the development of new compounds still represents a great challenge^35,36^.

In light of this, we sought to elucidate the mechanism of action of a series of crown ether compounds. We had previously described the biological activity of a number of these compounds and characterised them as an emerging group of antitumor agents^14,18,19^. Here we studied 16 structurally related adamantane substituted monoaza- (MAC) and diaza- (DAC) crown ether compounds. They differ in the lipophilicity, number of side chains, side chain length, and type of linkage of the side chain to aza-18-crown-6 (amine *vs*. amide linkage). We tested their anti-proliferative activity in an MDR cell line model (A2780/Adr and the parental A2780 cell line). DAC compounds effectively inhibit growth in both cell lines. This is in line with previously published activities on other tumour cell lines and could be related to crown ether affinity for alkali cations (especially for potassium ion), better extraction capabilities and lipophilicity^19,20^. For most compounds, A2780/Adr were slightly more resistant as compared to A2780.

We further tested the ability of crown ethers to modulate the functional activity of P-gp. Crown ether treatment resulted in decreased Rho123 efflux from both MDR cell models used (A2780/Adr and MDCKII-MDR1), indicating their inhibitory activity towards P-gp. Among diaza-crown derivatives, compound DAC-2Amide demonstrated the most prominent inhibitory activity, followed by DAC-3Amide, -1Amide and -4Amide. When compared to inhibitory potentials of different commercially available P-gp inhibitors, DAC-2Amide activity was stronger than verapamil and comparable to cyclosporine A (first generation inhibitors). However, it was significantly less potent than PSC-833 (second generation inhibitor). These results were corroborated by calcein accumulation assay. Calcein accumulation should not be affected by compounds that alter mitochondrial or plasma membrane potential, as compared to Rho123, which is a positively charged molecule^37^.

Compounds with amide sidearms caused stronger P-gp inhibition than their structurally related analogues with an amine bond, which is contrary to anti-proliferative activity. The presence of a carbonyl group adjacent to nitrogen of the macro-ring rigidifies the structure due to amide group resonance properties^18,38^. This points to the importance of the rigidity of the macro-ring structure for P-gp inhibition. Moreover, the results uncover the importance of side-chain length, whereby the compound with two carbon atoms in the linkage has the best inhibitory activity (i.e. DAC-2Amide). Interestingly, ion complexation and/or the transporting ability of DACs with amine linkage positively correlate with the cytotoxic activity, most probably as a consequence of disturbed ion homeostasis. However, compounds with low ion complexation properties tend to be less toxic, but have superior P-gp inhibitory activity.

Furthermore, monoaza- crown ethers showed inhibitory potential towards P-gp as well, but they were statistically less active compared to verapamil. In contrast to DACs, the activity of MACs did not correlate with the type of linkage to adamantane moiety, but instead with their lipophilicity.

It is generally accepted that the lipophilicity is one of the most important physicochemical properties used in drug candidate selection. Various SAR and QSAR studies revealed that high lipophilicity seems to be a general prerequisite for high P-gp inhibitory potential^39,40^. Therefore, compounds with significant P-gp inhibitory activity should have low molecular weight and optimal lipophilicity to show a low chance of failure in the drug discovery pipeline^2,40–43^.

Compound lipophilicity is described with its characteristic logP value. We previously showed that this is the most important molecular descriptor for biological activity of 18-crown-6 ethers, the optimal logP being 5.5 (Ghose-Crippen ALOGP estimate)^19^. Therefore, we calculated the correlation of logP values for the whole set of compounds to relative P-gp inhibitory activity in order to determine its importance. The relationship between the two variables was polynomial (for all compounds), whereby the predicted activity peak was ALOGP ≈ 4. Experiments show that DAC-2Amide and -3Amide have the best inhibitory activity. Calculations show that they have near optimal ALOGP ≈ 4, while their inhibitory activity is considerably better than what both polynomial and linear models predict. This greater efficiency suggests that in order to inhibit P-gp effectively, crown ethers require an optimal lipophilicity to enter the cell membrane and an additional conformational plasticity to access P-gp binding sites. The most important of these requirements are two lipophilic side chains of two C atoms, linked to aza-18-crown-6 by an amide bond.

We further investigated whether co-treatment with the most potent crown ethers increased sensitization of resistant A2780/Adr ovarian carcinoma cells to paclitaxel (PTX). The parent A2780 cells were found to be very sensitive to PTX treatment (IC_50_ = 2.9 nM), while the resistant cells remained completely unaffected at the same concentration range (IC_50_ > 100 nM). Indeed, the co-treatment of A2780/Adr cells with the non-toxic concentrations of DAC-2Amide, -3Amide or -4Amide and PTX resulted in a significant and dose-dependent cell sensitization to PTX. DAC-2Amide was the most effective and already at 1.5 µM sensitized cells towards PTX to a similar extent as 10 µM verapamil. This is in line with Rho123 efflux results, which showed DAC-2Amide to be more effective than verapamil. Similar results were obtained by co-treatment of A2780/Adr cells with crown ethers and another chemotherapeutic – adriamycin. Given that the mechanism of action of adriamycin differs from that of PTX, the observed crown ether effect appear to be independent of the type of chemotherapeutic used. Besides the influence on viability, combined treatment of resistant cells with DAC-2Amide or -3Amide with PTX induced a notable G2/M arrest, along with an increase in the apoptotic cell population. These effects do not appear in the individual treatments with the same concentrations of tested compounds. The increase in the mitotic population and activation of programmed cell death is a typical consequence of PTX activity. This observation substantiates the conclusion that crown ethers modulate P-gp function by inhibiting the efflux of PTX and allowing its antitumor activity. A comparable effect was obtained by the combination of PTX and verapamil. Finally, we can conclude that co-treatment with crown ethers DAC-2Amide or DAC-3Amide leads to the reversal of resistance due to decreased P-gp mediated efflux of chemotherapeutics.

The exact mechanisms of action of most P-gp modulators are still being investigated. Nevertheless, the modulators can be divided according to their molecular effects: modulators which inhibit P-gp activity, modulators which downregulate P-gp expression, or modulators which both inhibit P-gp activity and downregulate its expression. Further, the inhibitors can be described as competitive, allosteric or inhibitors of P-gp ATPase activity. Competitive inhibitors saturate the substrate binding site and can lead to undesirable induction of P-gp expression. On the other hand, non-competitive inhibitors negatively affect the activity of P-gp through allosteric modulation. In addition, some compounds act by blocking ATP binding to the protein and disable its ATP-driven translocation activity^25,44^.

In order to further examine inhibitory mechanism of the most active compounds, we used two different functional assays. First we employed UIC2 shift assay to inspect whether crown ethers affect P-gp conformation, and to establish the nature of the interaction or modulation we complemented it with an ATPase assay^24,45^. UIC2 antibody binds to P-gp before the last step of the substrate transport, inhibiting the transition into the outward-open conformation and release of the substrate^46^. Thus, P-gp substrates and modulators that reach the internal cavity render the transporter accessible to the UIC2 antibody, which is detected as increased binding. A similar effect is also elicited by ATP-depleting agents, or when both nucleotide-binding sites are inactivated by mutations^26,44,45^. Our results show increased binding of UIC2 antibody to P-gp upon the treatment with PTX, confirming it is a substrate of the transporter. On the other hand, DAC-2Amide, -3Amide, -4Amide (1 µM) and verapamil (50 µM) did not induce UIC2 shift, suggesting that they are probably not P-gp substrates. Nevertheless, it has been previously described that some P-gp substrates also fail to alter UIC2 binding depending on their stoichiometry and ability to stimulate P-gp ATPase activity (e.g. etoposide and colchicine)^24^. This might be related to the relatively low ability of these drugs to stimulate P-gp ATPase activity. However, verapamil is known to stimulate P-gp ATPase activity. Previous studies demonstrated a positive UIC2 shift elicited by verapamil at 50 µM concentrations^45^. Although somewhat unexpected, the absence of UIC2 shift after verapamil treatment (50 µM) can be correlated to its specific mechanism of modulation, as it has been considered both a competitive and non-competitive inhibitor of P-gp^26,45,47,48^. Moreover, in addition to interacting with substrate binding site, verapamil was shown to compete with the catalytic domain of the P-gp ATPase^31,47,49^. Our results show decreased UIC2 binding to P-gp upon the treatment with 10 µM verapamil or DAC-4Amide. These results indicate conformational change of P-gp similar to the one observed after the treatment with non-competitive modulators or P-gp ATPase blocker sodium orthovanadate^25,26,48^.

Substrate-stimulated ATPase activity of P-gp implies a close link between ATP hydrolysis and drug transport. Still, the molecular mechanisms linking ATP binding and/or hydrolysis to conformational changes of P-gp are not fully understood. Moreover, recent study by Alam *et al*. showed that P-gp can go through ATP hydrolysis cycle even when UIC2 is bound to it^46^. Nevertheless, compounds that stimulate ATPase activity are generally considered as substrates for the transporter. However, both substrates and inhibitors can activate ATPase. For example, verapamil, in addition to being an inhibitor of P-gp, stimulates P-gp ATPase activity^27^.

Currently available data suggest that the binding of P-gp substrates and modulators is a complex process that might include more than one substrate binding site. These different sites appear to exhibit various mutual relationships in the context of drug binding. They can be either independent or in exclusive interaction. Additionally, they can exhibit a partial negative interaction^48^. The complexity of P-gp binding sites could explain the observed ATPase activation by 1 µM concentration of DAC-2Amide and -3Amide. This indicates that they could act as P-gp substrates at certain concentrations, in spite of the absence of UIC2 shift (similarly to verapamil). The observed effect could also be due to the difference in binding affinity between presumed exclusive binding sites for the substrate and modulator^50^.

As previously reported, the effect on P-gp ATPase activity can be different, depending on substrate concentrations used in the experiment. Specifically, the increase of ATPase activity was observed at lower compound concentrations, and a decrease at higher concentrations. In addition, the majority of compounds classified as inhibitors (except cyclosporine A) do activate P-gp ATPase^27,50^. We observed a similar trend upon treatment with effective crown ether compounds. Both compounds inhibit the ATPase activity in a concentration-dependent manner, whereby ATPase activity was decreased below basal activity upon treatment with high compound concentrations. These results suggest that the compounds favour inhibition versus activation of ATPase in a stoichiometric manner. On the other hand, P-gp ATPase inhibition can be achieved indirectly after the collapse of optimal membrane properties necessary for ABC transporter activity.

P-gp is a transmembrane protein and thus its activity highly depends on biochemical and biophysical properties of the membrane. Alterations of plasma membrane lipids and their environment can affect a number of important carrier-mediated processes, such as P-gp substrate affinity, ATP binding and hydrolysis^51–53^. Moreover, most of the MDR reversing compounds are amphiphilic and influence biophysical properties of lipid bilayers by altering membrane fluidity and increasing its permeability (e.g. promethazine, progesterone, quinine and verapamil)^31,54,55^. Therefore, many putative substrates and chemosensitisers can permeabilize the cell membrane non-specifically, rather than directly interacting with P-gp^56^. Hence, the interaction of modulators with the lipid phase of the cell membrane seems to be as important for MDR reversal as their interaction with MDR proteins. In spite of being membrane-active compounds, our results point to a clear interaction between crown ethers and P-gp. Of note, crown-ethers activate P-gp ATPase and specifically sensitise MDR cells to paclitaxel at low and non-toxic concentrations. This suggests that the crown ether interaction site with P-gp either overlaps with the substrate and/or ATPase binding site, or it is linked to it in a negative allosteric fashion. Additionally, the effect of crown ethers on plasma membrane disruption becomes prominent only at higher compound concentrations.

As previously shown, the PI3K/Akt pathway is involved in modulating P-gp-mediated MDR and its inhibition can reverse this type of MDR^28^. Additionally, the expression of the Raf/MEK/ERK pathway modulates the expression of drug pumps^29^. Therefore, we analysed whether the tested compounds affect P-gp expression through PI3K/Akt or MEK/ERK signalling. Although the tested compounds did affect Akt signalling pathway, this did not ultimately alter P-gp expression and is probably not the underlying mechanism of action.

In addition, long term MDR cell treatment with low dose of crown ethers did not demonstrate any change in P-gp expression on the level of the whole cell, nor at the plasma membrane. Nevertheless, we observed a slight decrease of Rho123 efflux in cells treated with DAC-2Amide, indicating that extended exposure affects P-gp activity. We cannot exclude a possibility that crown ethers might disturb protein recycling or trafficking, or have a negative influence on biophysical properties of the plasma membrane.

In conclusion, we demonstrate a novel use of crown ethers for inhibition of P-gp and reversal of MDR phenotype. For achieving the best inhibitory activity, crown ethers need to have optimal lipophilicity and structural features to enter the membrane and interact with P-gp. The most potent compounds in our study have ALOGP of around 4, lipophilic side chains of two C atoms, and are linked to aza-18-crown-6 by an amide bond. They do not change the P-gp expression, nor its localisation. Their effect is related to their ability to modulate P-gp ATPase activity. At low concentrations they could act as substrates and inhibitors, while at higher concentrations they probably change the membrane integrity necessary for proper P-gp function. Their potential should be further investigated on *in vivo* models, while design of novel compounds having MDR-inhibitory activity should comply with molecular characteristics described in our study.

## Methods

### Synthesis

Procedures and methods for crown ether synthesis are shown in the Supplementary Information.

### Cell culturing

Human ovarian carcinoma cell line A2780 (ECACC cat. no. 93112519) and its adriamycin resistant counterpart A2780/Adr (ECACC cat. no. 93112520) were obtained from The European Collection of Authenticated Cell Cultures. A2780/Adr cell line was developed by adriamycin treatment of parental A2780 cell line. In order to retain resistance, 0.1 µM adriamycin was added in the cell culture once a week.

Wild-type Madin-Darby canine kidney cells type II (MDCKII-WT) and stable MDCKII transfectants overexpressing human P-glycoprotein (MDCKII-MDR1), were kindly provided by Drs. A. Schinkel and P. Borst, The Netherlands Cancer Institute, Amsterdam.

Cells were grown in RPMI-1640 (A2780) or DMEM (MDCK-II) medium with the addition of 10% fetal bovine serum (FBS), 2 mM L-glutamine, 100 U/ml penicillin and 100 µg/ml streptomycin, and cultured as monolayers at 37 °C in a humidified atmosphere with 5% CO_2_.

### Cell viability assay

Cell viability assay was performed as described previously^18,19^. Cells were seeded at 2 × 10^4^ to 4 × 10^4^ cells/well (depending on the doubling time of a specific cell line) in a standard 96-well microtiter plates and left to attach for 24 h. Next day, test compounds were added in five serial 10-fold dilutions alone or in combination with paclitaxel or adriamycin. The final concentration of DMSO was <0.2% which was non-toxic to cells. The cell growth rate was evaluated after 72 hours of incubation, using MTT assay. Obtained results are expressed as IC_50_ value which stands for the concentration of compound necessary for 50% of growth inhibition as compared to non-treated control cells. The IC_50_ values for each compound are calculated from concentration-response curves using linear regression analysis.

### Rhodamine 123 efflux assay

The cells were collected and incubated at 2 × 10^5^ cells in complete cell culture medium (see above) in the presence or absence of 0.5 µM Rhodamine 123 (Rho123; Sigma-Aldrich) in the dark at 37 °C. After 30 minutes of incubation, cells were washed thoroughly with ice cold PBS and subsequently incubated in Rho123-free medium with the addition of tested compounds in the dark for 45 min at 37 °C. Cells were then washed thoroughly with ice cold PBS and analysed by flow cytometry. The median fluorescence intensity (MFI) of Rho 123 was measured on a BD FACSCalibur flow cytometer (Becton Dickinson) with excitation at 488 nm and the emitted fluorescence was collected through a 530 nm bandpass filter. Data analysis was performed using FlowJo software (TreeStar Inc.). For Supplementary Fig S13C, the protocol was modified so that in the second step no compounds were added. This allowed to evaluate the effect of long-term exposure to crown ethers.

### Calcein accumulation assay

For the calcein accumulation assay, 3 × 10^4^ cells were seeded into 96-well microtiter plates and left to attach. Next day, cells were washed with PBS and incubated with 200 µL of RPMI-1640 medium (without FBS) containing 0.25 µM calcein-AM (Thermo Fisher Scientific) and 10 µM of tested compound. After 60 minutes of incubation, media was discarded and cells were washed with PBS. After last wash, the accumulated Calcein within the cells was released into solution by treatment with 200 µL of 0.2% Triton X-100. Calcein fluorescence was measured on a microplate fluorimeter reader (Tecan) with excitation beam of 485 nm and the emitted fluorescence was collected at 530 nm.

### Cell cycle analysis

For the analysis of cell cycle, A2780/Adr cells were seeded at 2 × 10^5^ cells per well into 6-well plates and left to attach. The next day, cells were treated with the tested compounds alone or in combination at indicated concentrations, and incubated for 48 hours at 37 °C. All cells were collected (attached and floating), washed by PBS, fixed with ice-cold 70% ethanol and stored overnight at −20 °C. Immediately before analysis, the cells were washed thoroughly with PBS and incubated with 0.1 μg/μl of RNAse A for 15 minutes at 37 °C. Finally, cells were stained with 50 μg/ml of propidium iodide (PI) for 30 minutes on ice. Stained cells were analysed on a BD FACSCalibur flow cytometer (20000 events per sample were collected). The percentage of cells in each cell cycle phase was determined using cell cycle module within FlowJo software.

### Annexin V assay

Cells were seeded onto 6-well plates at a density of 2 × 10^5^ cells/well and left to attach overnight. Next day, cells were treated with compounds alone or in combinations at indicated concentrations. After 48 hours of incubation, all cells were collected (attached and floating), washed thoroughly with PBS, and re-suspended in the Annexin binding buffer (10 mM HEPES, 140 mM NaCl, 2.5 mM CaCl_2_, pH = 7.4) containing Annexin V FITC conjugate (BD Pharmigen) and 4 mg/mL 7-actinomycin D (7-AAD, Molecular Bioprobes, Invitrogen). The cells were analyzed on BD FACSCalibur flow cytometer (10000 events were collected). Fluorescence compensation and analysis was performed with FlowJo software. Both Annexin V positive and Annexin V/7-AAD double positive cells were determined and the percentage of total apoptotic cells was calculated.

### Immunophenotyping

Cells were seeded onto 6-well plates at a density of 1.3 × 10^5^ cells/well and left to attach overnight. Next day, cells were treated with 2 µM concentration of compounds. After 72 hours of incubation, only attached cells were collected and washed with blocking solution (2% BSA in PBS). Cells were then incubated for 30 minutes with anti-P-gp primary antibody in blocking solution (1:250, clone 4E3, Biolegend). After washing with blocking solution, cells were incubated with the corresponding goat anti-mouse Alexa-fluor 488 secondary antibody for 30 minutes (1:300, Thermo Fisher Scientific). Cells were washed with PBS twice and analysed by flow cytometry. The median fluorescence intensity (MFI) was measured on a BD FACSCalibur flow cytometer (Becton Dickinson) with excitation at 488 nm and the emitted fluorescence was collected through a 530 nm bandpass filter. Data analysis was performed using FlowJo software (TreeStar Inc.).

### UIC2 shift assay

Briefly, cells were collected by trypsinization and samples containing 2 × 10^5^ cells/vial were washed with PBS. Cells were resuspended in blocking buffer (PBS with 2% BSA and 5.5 mM glucose) and allowed to equilibrate for 10 minutes at 37 °C. Subsequently, cells were treated with freshly prepared compound solutions in blocking buffer and incubated for an additional 10 minutes at 37 °C. Finally, mouse monoclonal anti-MDR1 antibody (clone UIC2, azide free, Millipore) at 1:800 dilution was added to the samples and incubated for 30 minutes at 37 °C. Cells were then thoroughly washed with ice cold PBS and incubated with goat anti-mouse Alexa fluor 488 secondary antibody (1:250, Thermo Fisher Scientific) for 30 minutes at room temperature in the dark. Prior the analysis, cells were again thoroughly washed with ice cold PBS and kept on ice until analysis by flow cytometry (BD FACSCalibur, Becton Dickinson, 10000 cells were collected). Excitation was done by an argon ion laser operating at 488 nm and the emitted fluorescence was collected through a 530 nm pass filter. Data analysis was performed using FlowJo software.

### Protein extraction and Western blot

Cells were collected by trypsinization, pelleted and washed with PBS on ice. Pellets were lysed with RIPA buffer supplemented with complete protease inhibitor (Sigma-Aldrich, USA) and subsequently sonicated using Bioruptor sonicator (Diagenode). Total concentration of proteins in the obtained cell lysates was measured by Pierce BCA Protein Assay Kit (Thermo Fisher Scientific) according to the recommendations from the manufacturer. 50 µg of protein per sample were loaded on a gel, separated using SDS-PAGE and transferred to PVDF membrane (Carl Roth, Germany). After protein transfer, membranes were stained using naphthol blue solution (0,1% naphthol blue in 10% methanol, 2% acetic acid) to visualise all proteins that was used to confirm equal sample loading. Membranes were incubated in blocking solution (5% non-fat milk in TBST) for 20 minutes at room temperature. Membranes were incubated with primary antibody at 4 °C overnight. Primary antibodies used were: p-AKT1 (1:2000, Ser473, clone D9E, Cell Signaling Technologies); AKT1 (1:300, clone 40D4, Cell Signalling Technologies), p-ERK1 (1:1000, clone E-4, Santa Cruz Biotechnology), ERK1 (1:1000, clone K-23, Santa Cruz Biotechnology) and ABCB1 (P-gp or Mdr-1, 1:100, clone G-1, Santa Cruz Biotechnology). After washing the membranes with TBST, membranes were incubated with HRP conjugated secondary antibody (anti-mouse IgG-HRP, 1:10000, GE Healthcare or anti-rabbit IgG-HRP, 1:5000, Bio-Rad) for 2 hours at room temperature and subsequently washed again with TBST before detection of signal by Western Lightning Plus-ECL reagent (Perkin-Elmer, USA). Emitted signal from membranes was collected and visualized with UVITEC imaging system (Cleaver Scientific Ltd) and images were prepared in Photoshop CS2 (Adobe).

### Immunofluorescence

Cells were seeded onto poly-L-Lysine treated glass cover slides, left to attach and next day treated with compounds as indicated. To stain for total P-gp protein we used anti-P-gp antibody (clone G1, SCBT) and the following protocol: After 72 hours of incubation slides were fixated with 4% PFA for 10 minutes and then permeabilised with blocking solution (2% BSA, 0.05% Tween-20 in PBS) for another 10 minutes. Slides were then incubated with 1:100 antibody dilution in blocking solution for 30 minutes at room temperature (RT). Slides were then washed and incubated with complementary goat anti-mouse Alexa Fluor 488 secondary antibody (Thermo Fisher Scientific) diluted 1:300 in blocking solution at RT. After incubation for 30 minutes, slides were washed and incubated with phalloidin-Alexa fluor 594 (1:40, Invitrogen) for 30 minutes at RT. In the end, slides were extensively washed with blocking solution, and in the last washing step 100 ng/mL of DAPI was used as a counter stain. Slides were then mounted using Fluoromount antifade reagent (Sigma-Aldrich). To stain just plasma membrane bound P-gp, we used another anti-P-gp antibody (clone 4E3, BioLegend) that recognizes the extracellular epitope of the protein. Similar staining protocol was followed except that the anti-P-gp (4E3) and complementary secondary antibody (same as above) were diluted (1:500 and 1:300, respectively) in blocking solution without Tween-20. Cells were fixated after the incubation with secondary antibody, permeabilized and phalloidin-Alexa fluor 594 was added as noted above for G1 antibody. Slides were counterstained with DAPI and mounted onto slides using Fluoromount antifade reaget. Images were collected on a Leica SP8 confocal microscope, processed in ImageJ software and prepared for publication in Illustrator CS2 (Adobe).

### ATPase assay

P-gp efflux activity is ATP-dependent, whereby hydrolysis of each ATP molecule yields inorganic phosphate (Pi). Therefore, the amount of free Pi is directly proportional to the activity of P-gp and can be measured colorimetrically. P-gp ATPase activity was measured using SB-MDR1-PREDEASY^TM^ ATPase Kit (Solvo Biotechnology, Budapest, Hungary) according to the manufacturer’s instructions and methods described by Sarkadi *et al*.^57^. Briefly, membrane vesicles containing P-gp transporter, purified from insect cells, were diluted with assay mix and loaded into a 96-well microplate. One microliter of the tested compound dissolved in DMSO with or without Na-orthovanadate (600 mM) was added to the membrane suspension. The same volume of DMSO was added to the control wells. The mixtures were pre-incubated at 37 °C for 10 minutes and the reaction was started by addition of 10 μL Mg-ATP (200 mM). After 10 minutes of incubation at 37 °C, the inorganic phosphate (Pi) released was determined colorimetrically, measuring the absorbance at 600 nm. For the inhibition experiment, ATPase assays for test compounds were performed in the presence of 40 μM verapamil, which is used to stimulate the ATPase. The data collected are represented as a percentage of difference between maximal ATPase activity (100%) and basal ATPase activity (0%), and curves obtained for the test compound can be described as an ATPase activator or inhibitor. The assay was performed in duplicates and repeated three times.

### Statistics

All graphics with error bars are presented as mean ± s.d. (except in Supplementary Figs S4 and S5 where only mean is shown). To determine statistical significance between samples, one-way ANOVA with Dunnett’s post-hoc test (Fig. 4a, Supplementary Fig S13A,C) or with Tukey’s post-hoc test (Figs 2a–d, 3d,e, Supplementary Fig. S6) was used. Statistical calculations were performed in GraphPad Prism and generation of graphics in Excel and Adobe Illustrator CS2 (NS – non-significant; *p < 0.05, **p < 0.01 and ***p < 0.001).

## Electronic supplementary material

Supplementary information

## Data Availability

All data generated or analysed during this study are included in this published article (and its Supplementary Information files).
