# Supplementary material for: Crown ethers reverse P-glycoprotein-mediated multidrug resistance in cancer cells
Source: Sci Rep. 2018 Sep 27;8:14467. doi: 10.1038/s41598-018-32770-y (PMC6160470; doi:10.1038/s41598-018-32770-y)
Supplement: Supplementary file 1 — Supplementary information [file 41598_2018_32770_MOESM1_ESM.pdf]

Crown ethers reverse P-glycoprotein-mediated multidrug resistance in cancer cells

Iva Guberović# [1],&, Marko Marjanović#[1], Marija Mioč[1], Katja Ester[1], Irena Martin-Kleiner[1], Tatjana Šumanovac Ramljak[2], Kata Mlinarić-Majerski[2], Marijeta Kralj[1],*

[1] Division of Molecular Medicine, Ruđer Bošković Institute, Bijenička cesta 54, 10 000 Zagreb, Croatia

[2] Department of Organic Chemistry and Biochemistry, Ruđer Bošković Institute, Bijenička cesta 54, 10000 Zagreb, Croatia

*marijeta.kralj@irb.hr

# - These authors contributed equally to this work

& - Current address: Josep Carreras Leukaemia Research Institute, Campus ICO-Germans Trias i Pujol-Universitat Autònoma de Barcelona, 08916 Badalona, Spain

Table of contents:

1. Synthesis and characterization of compounds (Supplementary Figures S1 and S2) S2

2. Extraction experiments (Supplementary Tables S1 and S2) S6

3. Evaluation of MDR phenotype (Supplementary Figures S3 and S4) S9

4. Cell growth inhibition by crown ethers (Supplementary Figures S5) S11

5. Calcein accumulation assay (Supplementary Figure S6) S13

6. Sensitization of P-gp overexpressing cells towards paclitaxel and doxorubicin (Supplementary Table S3 and Figures S7-S12) S14

7. Effects of long-term exposure of crown ethers on P-gp expression and functionality (Supplementary Figure S13) S20

8. Full length blot images corresponding to Figure 5. (Supplementary Figure S14) S21

Synthesis and characterization of compounds

General

IR spectra were recorded on a FT-IR-ABB Bomem MB 102 spectrophotometer in KBr. ^{1}H and ^{13}C NMR spectra were recorded on a Bruker AV- 300 or 600 MHz. The NMR spectra were taken in $CDCl_3$ using TMS as a reference and chemical shifts are reported in ppm. HRMS were obtained on an Applied Biosystems 4800 Plus MALDI TOF/TOF instrument (AB, Foster City, CA). Solvents for chromatography were of HPLC purity. All compounds were routinely checked by aluminum oxide TLC-plates. Monoaza-18-crown-6 and 4,13-diaza-18-crown-6,[1,2] as well as monoaza-crown ethers: MAC-2, MAC-2Amide, MAC-3, MAC-3Amide,[3] and diaza-crown ethers DAC-1, DAC-1Amide, DAC-2, DAC-2Amide, DAC-3, DAC-3Amide [4-6,] were prepared according literature procedure. Adamantane compounds, precursors for the synthesis of mona- and diaza-crown ethers were prepared according to the literature procedures.[3,6,7-11] Synthesis of new compounds: MAC-1, MAC-1Amide, MAC-4, MAC-4Amide, DAC-4, and DAC-4Amide are described here.

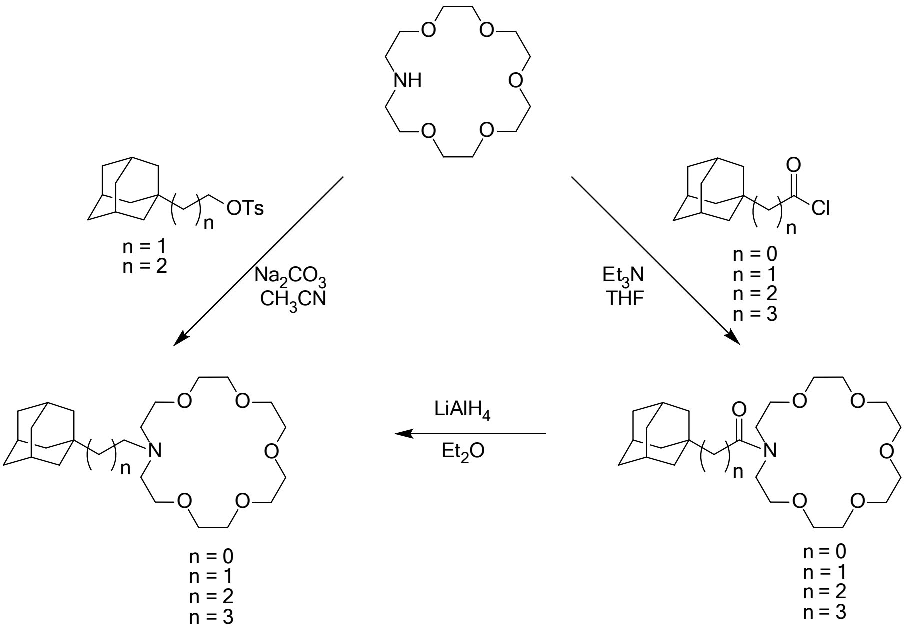

Supplementary Figure S1. Synthesis of monoaza-crown ethers

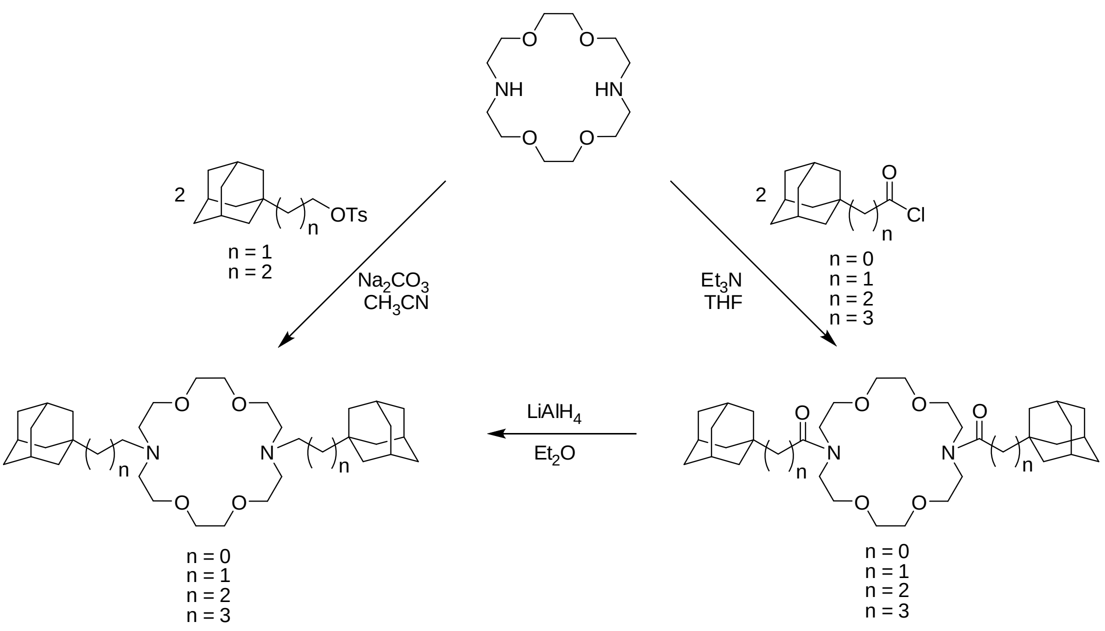

Supplementary Figure S2. Synthesis of diaza-crown ethers

General procedure for the preparation of adamantane functionalized monoaza- and diaza-crown ethers by coupling reactions of crown ether and acyl chloride

In a reaction vessel under a stream of N_2, one equivalent of monoaza-18-crown-6 or diaza-18-crown-6 was dissolved in THF and 2.5 or 5 equivalents of triethylamine were added. The resulting mixture was stirred at ambient temperature for 10 min, and a solution of corresponding acyl chloride (one or two equivalents) was added. The reaction mixture was stirred at ambient temperature for additional 24 hours, filtered and the filtrate was evaporated under reduced pressure. The solid residue was suspended in CH_2Cl_2, washed with saturated solution of NaCl and dried over anhydrous $MgSO_4$. After removal of the solvent, under reduced pressure, the oily product was obtained. The product was additionally purified by column chromatography.

N-(1-adamantanoyl)-aza-18-crown-6 (**MAC-1Amide**)

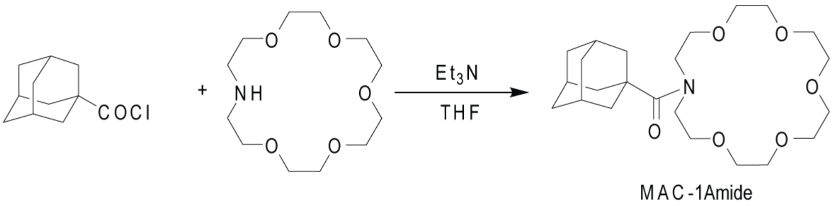

By following the general procedure, crown ether **MAC-1Amide** was obtained by reaction of monoaza-18-crown-6 (0.526 g, 2.0 mmol) and 1-adamantanoyl chloride (0,400 g, 2.0 mmol). The crude product was purified via column chromatography on Al_2O_3 (act. II-III) using 0→2% MeOH in CH_2Cl_2 as an eluent, thereby affording 0.514 g (61%) of product **MAC-1Amide** as a colorless oil.

^{1}H NMR ($CDCl_3$) δ/ppm: 1.72 (br. s, 6H), 2.00 (br. s, 6H), 2.04 (br. s, 3H), 3.60–3.70 (m, 24 H). ^{13}C NMR ($CDCl_3$) δ/ppm: 28.41 (d, 3C), 36.48 (t, 3C), 39.04 (t, 3C), 41.95 (s, 1C), 48.63

(t, 2C), 69.68 (t, 2C), 70.43 (t, 2C), 70.46 (t, 2C), 70.51 (t, 2C), 70.64 (t, 2C), 176.92 (s, 1C). IR (KBr) ν_{max}/cm^{-1}: 2905 (s), 1620 (s), 1452 (m), 1404 (m), 1350 (m), 1294 (m), 1230 (m), 1121 (s), 943 (w). HRMS, calculated for [$C_{23}H_{39}NO_6$ + H^+]: 426.2850; observed: 426.2851.

N-[1-oxo-4-(1-adamantyl)butyl]-aza-18-crown-6 (**MAC-4Amide**)

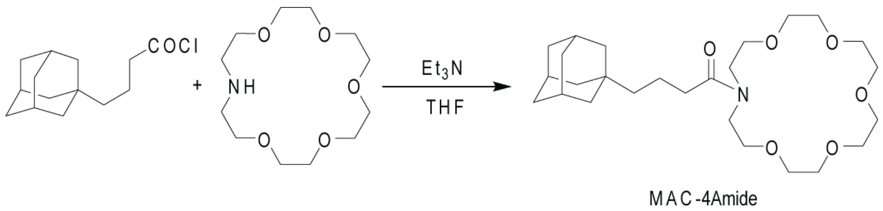

By following the general procedure, crown ether **MAC-4Amide** was obtained by reaction of monoaza-18-crown-6 (0.132 g, 0.5 mmol) and 1-(chlorobutanoyl)adamantane (0,12 g, 0.5 mmol). The crude product was purified via column chromatography on Al_2O_3 (act. II-III) using 0→2% MeOH in CH_2Cl_2 as an eluent, thereby affording 0.055 g (24%) of product **MAC-4Amide** as a colorless oil.

^{1}H NMR ($CDCl_3$) δ/ppm: 1.02–1.08 (m, 2H), 1.48 (br. s, 6H), 1.55-1.65 (m, 5H), 1.65–1.72 (m, 3 H), 1.93 (br. s, 3H), 2.20–2.32 (m, 2H), 3.55–3.65 (m, 24H). ^{13}C NMR ($CDCl_3$) δ/ppm: 18.46 (t, 1C), 28.60 (d, 3C), 32.19 (s, 1C), 33.85 (t, 1C) 37.11 (t, 3C), 42.27 (t, 3C), 44.31 (t, 1C), 46.73 (t, 1C), 46.80 (t, 1C), 69.38 (t, 1C), 69.94 (t, 1C), 70.22 (t, 1C), 70.43 (t, 3C), 70.53 (t, 1C), 70.58 (t, 1C), 70.65 (t, 1C), 70.68 (t, 1C). IR (KBr) ν_{max}/cm^{-1} : 2901 (s), 1647 (m), 1450 (w), 1352 (w), 1121 (m). HRMS, calculated for [$C_{26}H_{45}NO_6$ + H^+]: 468.3319; observed: 468.3336.

N,N'-bis[1-oxo-4-(1-adamantyl)butyl]-4,13-diaza-18-crown-6 (**DAC-4Amide**)

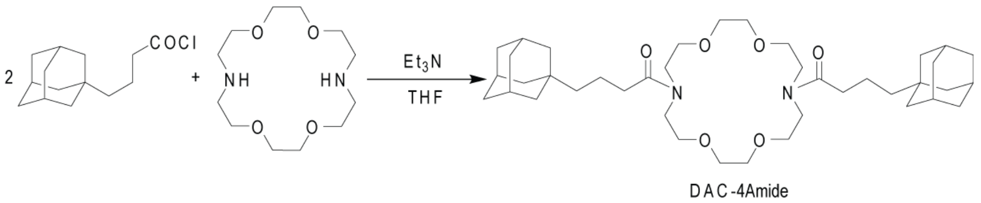

By following the general procedure, crown ether **DAC-4Amide** was obtained via reaction of diaza-18-crown-**6** (0.144 g, 0.55 mmol) and 1-(2-chlorobutanoyl)adamantane (0,270 g, 1.1 mmol) The crude product was purified via column chromatography on Al_2O_3 (act. II-III) using 0→2% MeOH in CH_2Cl_2 as an eluent, thereby affording 0.325 g (88%) of product **DAC-4Amide** as a colorless oil.

^{1}H NMR ($CDCl_3$) δ/ppm: 1.02–1.08 (m, 4H), 1.45–1.50 (m, 12H), 1.55–1.63 (m, 10 H), 1.66-1.72 (m, 6H), 1.92 (br. s, 6H), 2.20–2.30 (m, 4H), 3.55–3.70 (m, 24H). ^{13}C NMR ($CDCl_3$) δ/ppm: 18.45 (t, 1C), 18.50 (t, 1C), 28.61 (d, 6C), 32.21 (s, 2C), 33.84 (t, 1C), 33.91 (t, 1C), 37.11 (t, 6C), 42.29 (t, 6C), 44.32 (t, 2C), 46.76 (t, 1C), 46.91 (t, 1C), 48.61 (t, 1C), 48.74 (t, 1C), 69.42 (t, 1C), 69,94 (t, 2C),70.30 (t, 1C), 70.35 (t, 1C), 70.43 (t, 1C), 70.63 (t, 1C), 70.80 (t, 1C), 173.26 (s, 1C), 173.32 (s, 1C). IR (KBr) ν_{max}/cm^{-1} : 2901 (s), 2845 (m), 1647 (m),

1450 (w), 1119 (m). HRMS, calculated for [$C_{40}H_{66}N_2O_6$ + H^+]: 671.4994; observed: 671.4969.

General procedure for reduction with $LiAlH_4$

The solution of crown ether (1 equivalent) in Et_2O was added dropwise to the suspension of $LiAlH_4$ (3.3 or 6.6 equivalents) in Et_2O. The reaction mixture was heated at the reflux temperature for 7 hours. After cooling to the ambient temperature the excess of $LiAlH_4$ was destroyed by careful dropwise addition of water. The Et_2O solution was decanted, washed with a saturated solution of NaCl and dried over $MgSO_4$. After removal of the solvent under reduced pressure the oily product was obtained. The crude product was purified via column chromatography.

N-2-(1-adamantyl)ethyl-aza-18-crown-6 (**MAC-1**)

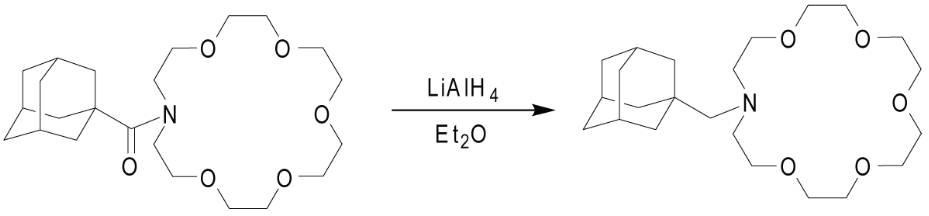

MAC-1Amide MAC-1

By following the general procedure, crown ether **MAC-1** was obtained by reaction of *N*-(1-adamantanoyl)-aza-18-crown-6 (**MAC-1Amide**) (0.069 g, 0.16 mmol). The crude product was purified via column chromatography on Al_2O_3 (act. II-III) using 0$\rightarrow$2% MeOH in CH_2Cl_2 as an eluent, thereby affording 0.040 g (62 %) of product **MAC-1** as a colorless oil.

^{1}H NMR ($CDCl_3$) δ/ppm: 1.45–1.50 (m, 6H), 1.65 (dd, 6H, J = 13.522 Hz i J = 11.861 Hz), 1.93 (br. s, 3H), 2.12 (br. s, 2 H), 2.75 (br. s, 4H), 3.55–3.70 (m, 20H). ^{13}C NMR ($CDCl_3$) δ/ppm: 28.54 (d, 3C), 29.55 (s, 1C), 35.01 (t, 1C), 37.27 (t, 3C), 41.14 (t, 3C), 57.27 (t, 2C), 70.34 (t, 1C), 70.53 (t, 2C), 70.61 (t, 2C), 70.84 (t, 3C), 70.91 (t, 2C). IR (KBr) ν_{max}/cm^{-1} : 2903 (s), 1655 (w), 1456 (w), 1354 (w), 1113 (m), 953 (w). HRMS, calculated for [$C_{23}H_{41}NO_5$ + H^+]: 412.3057; observed: 412.3054.

N-4-(1-adamantyl)butyl-aza-18-crown-6 (**MAC-4**)

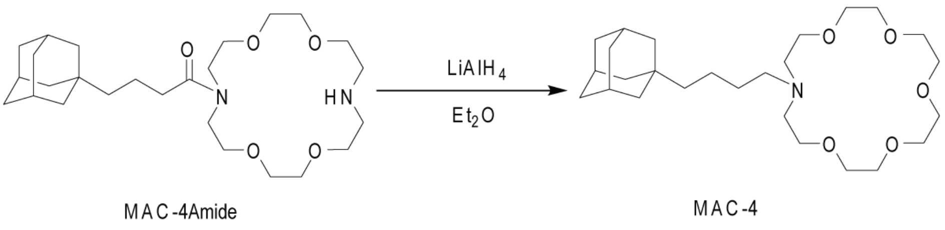

MAC-4Amide MAC-4

By following the general procedure, crown ether **MAC-4** was obtained by reaction of *N*-[1-oxo-4-(1-adamantyl)butyl]-aza-18-crown-6 (**MAC-4Amide**) (0.055 g, 0.12 mmol). The crude product was purified via column chromatography on Al_2O_3 (act. II-III) using 0→2% MeOH in CH_2Cl_2 as an eluent, thereby affording 0.032 g (59 %) of product **MAC-4** as a colorless oil.

^{1}H NMR ($CDCl_3$) δ/ppm: 1.00–1.05 (m, 2H), 1.15–1.25 (m, 2H), 1.35-1.40 (m, 2H), 1.43 (br. s, 6 H), 1.61 (d, 3H, J = 11.756 Hz), 1.69 (d, 3H, J = 11.756 Hz), 1.93 (br. s, 3H), 2.45–2.55 (m, 2H), 2.77 (br. s, 4H), 3.60–3.70 (m, 20H). ^{13}C NMR ($CDCl_3$) δ/ppm: 20.34 (t, 1C), 28.12 (t, 1C), 28.80 (d, 3C), 32.26 (s, 1C), 37.32 (t, 3C), 42.53 (t, 3C), 44.70 (t, 1C), 54.07 (t, 2C), 56.15 (t, 1C), 69.91 (t, 2C), 70.45 (t, 2C), 70.82 (t, 4C), 70.90 (t, 2C). IR (KBr) ν_{max}/cm^{-1} : 2901 (s), 1655 (w), 1458 (w), 1354 (w), 1101 (m), 955 (w). HRMS, calculated for [$C_{26}H_{47}NO_5 + H^+$]: 454.3527; observed: 454.3513.

N,N'-bis[4-(1-adamantyl)butyl]-4,13-diaza-18-crown-6 (**DAC-4**)

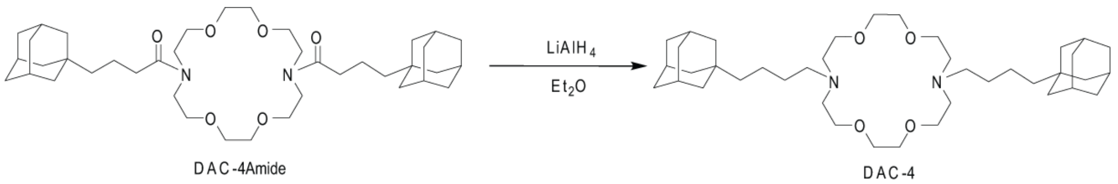

By following the general procedure, crown ether **DAC-4** was obtained by reaction of *N,N'*-bis[1-oxo-4-(1-adamantyl)butyl]-4,13-diaza-18-crown-6 (**DAC-4Amide**) (0.159 g, 0.24 mmol). The crude product was purified via column chromatography on Al_2O_3 (act. II-III) using 0→2% MeOH in CH_2Cl_2 as an eluent, thereby affording 0.060 g (39 %) of product **DAC-4** as a colorless oil.

^{1}H NMR ($CDCl_3$) δ/ppm: 0.90–1.05 (m, 4H), 1.12–1.22 (m, 4H), 1.35-1.46 (m, 16H), 1.55–1.72 (m, 12 H), 1.92 (br. s, 6H), 2.42–2.52 (m, 4H), 2.75–2.80 (m, 8H), 3.55–3.65 (m, 16H). ^{13}C NMR ($CDCl_3$) δ/ppm: 20.22 (t, 2C), 28.01 (t, 2C), 28.64 (d, 6C), 32.11 (s, 2C), 37.17 (t, 6C), 42.37 (t, 6C), 44.58 (t, 2C), 53.83 (t, 4C), 56.96 (t, 2C), 69.90 (t, 4C), 70.60 (t, 4C). IR (KBr) ν_{max}/cm^{-1} : 2901 (s), 2856 (m), 1655 (w), 1450 (w), 1099 (m). HRMS, calculated for [$C_{40}H_{70}N_2O_4 + H^+$]: 643.5407; observed: 643.5394.

Extraction experiments

Extraction experiments were performed as described previously,[3,6,12-14] by using 5×10^{-3} M $CHCl_3$ solution of the corresponding monoaza- or diaza-crown ethers and 5×10^{-3} M H_2O solution of alkali metal picrates. The picrate ion concentrations were determined by UV.

Supplementary Table S1. Extraction experiments of alkali picrates with $CHCl_3$ containing monoaza-crown ether

Compound	Percent of picrate extracted (%)[a]				
	Li+	Na+	K+	Rb+	Cs+
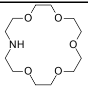 MAC	11,6 ± 0,3	35,5 ± 0,6	42,9 ± 0,6	37,0 ± 0,6	39,8 ± 0,5
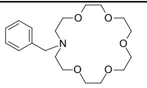 BMAC	0,2 ± 0,5	15,0 ± 0,8	51,3 ± 0,5	35,9 ± 0,4	18,4 ± 0,8
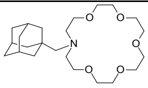 MAC-1	21,5 ± 0,8	27,8 ± 0,6	26,6 ± 0,4	19,3 ± 0,7	28,8 ± 1,0
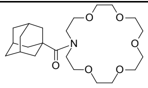 MAC-1Amide	0,1 ± 0,4	0,7 ± 0,8	0,5 ± 0,7	0,3 ± 0,5	0,5 ± 0,8
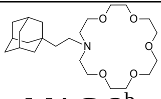 MAC-2[b]	2,2 ± 0,2	24,7 ± 0,7	52,8 ± 0,5	44,3 ± 0,6	28,3 ± 0,4
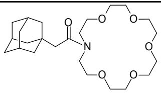 MAC-2Amide[b]	1,8 ± 0,3	0,4 ± 0,3	0,03 ± 0,1	1,0 ± 0,4	1,3 ± 0,4
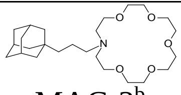 MAC-3[b]	0,8 ± 0,3	18,7 ± 0,3	34,9 ± 0,6	29,8 ± 0,3	21,6 ± 0,2
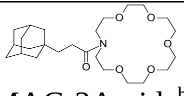 MAC-3Amide[b]	1,2 ± 0,5	3,9 ± 0,5	3,4 ± 0,4	3,7 ± 0,5	3,0 ± 0,6
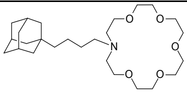 MAC-4	0,6 ± 0,7	1,2 ± 0,5	0,4 ± 0,5	0,7 ± 0,9	0,6 ± 0,7
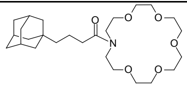 MAC-4Amide	0,4 ± 0,9	1,3 ± 0,8	1,7 ± 0,6	1,3 ± 0,8	0,6 ± 0,7

[a] Defined as percent of picrate extracted into organic phase. Each value is the average of five independent extraction experiments.
[b] Reference [3].

Supplementary Table S2. Extraction experiments of alkali picrates with $CHCl_3$ containing diaza-crown ethers

Compound	Percent of picrate extracted (%)[a]				
	Li^+	Na^+	K^+	Rb^+	Cs^+
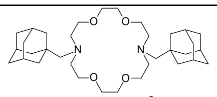 DAC-1[b]	27,1 ± 0,7	25,0 ± 0,6	44,1 ± 0,7	23,8 ± 0,7	26,5 ± 0,6
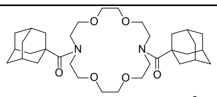 DAC-1Amide[b]	8,8 ± 0,3	5,2 ± 0,7	8,2 ± 0,3	6,0 ± 0,5	5,3 ± 0,6
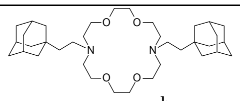 DAC-2[b]	22,5 ± 0,3	37,1 ± 0,7	57,0 ± 0,6	35,8 ± 0,8	39,4 ±0,8
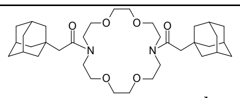 DAC-2Amide[b]	0,3 ± 0,3	0,6 ± 0,4	1,1 ± 0,4	0,5 ± 0,5	2,8 ± 0,3
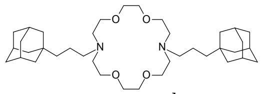 DAC-3[b]	27,1 ± 0,7	25,0 ± 0,6	44,1 ± 0,7	23,8 ± 0,7	26,5 ± 0,6
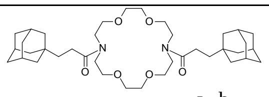 DAC-3Amide[b]	0,2 ± 0,1	1,0 ± 0,6	0,3 ± 0,1	0,2 ± 0,5	0,5 ± 0,4
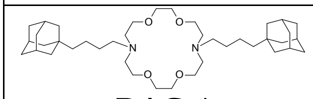 DAC-4	53,2 ± 0,4	40,1 ± 0,6	47,4 ± 0,5	37,2 ± 0,7	36,5 ± 0,5
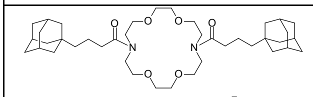 DAC-4Amide	0,7 ± 0,7	1,6 ± 0,6	3,6 ± 0,6	0,9 ± 0,7	0,5 ± 0,8
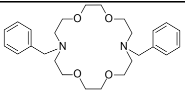 DBDAC	16,3 ± 0,8	17,4 ± 0,7	31,2 ± 0,6	7,5 ± 0,6	8,6 ±0,7
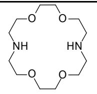 DAC	23,1 ± 0,5	20,8 ± 0,3	25,4 ± 0,7	10,3 ± 0,3	18,8 ±0,8

[a] Defined as percent of picrate extracted into organic phase. Each value is the average of five independent extraction experiments.
[b] Reference [6].

Evaluation of MDR phenotype

a

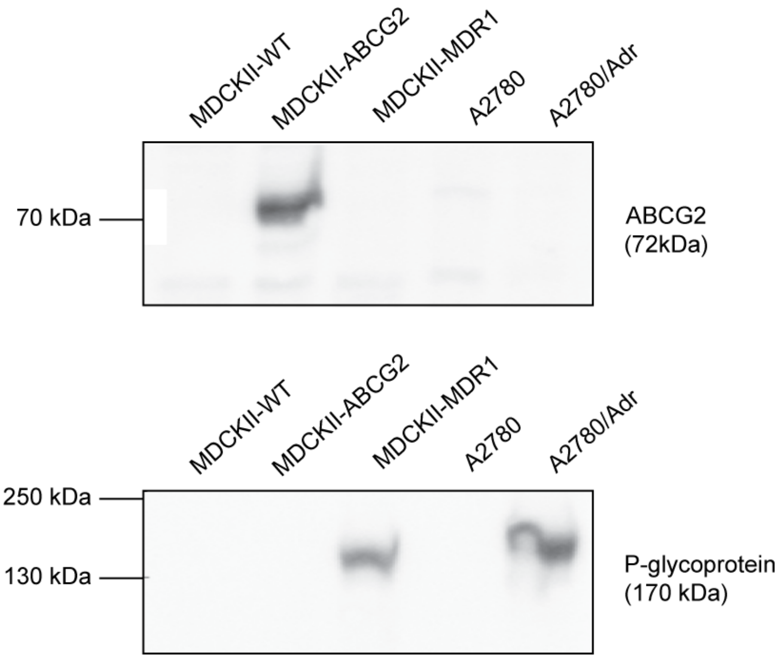

b

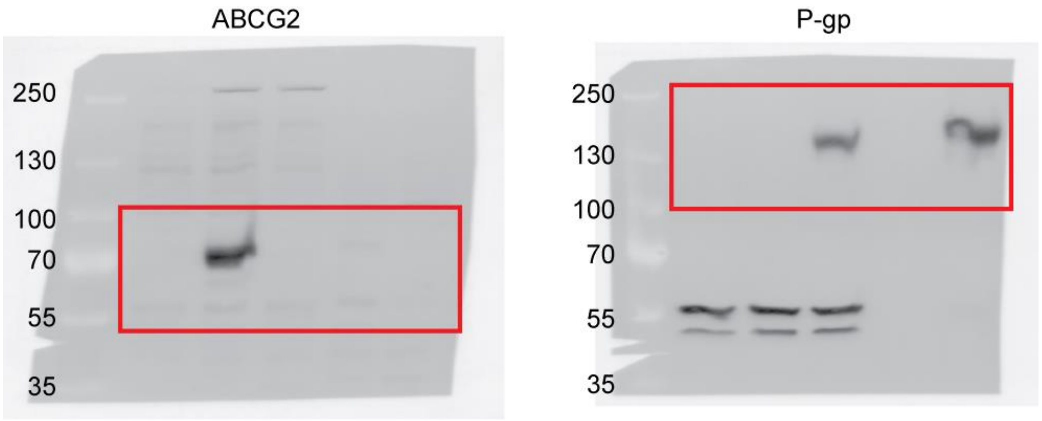

Supplementary Figure S3. Cell lines with MDR phenotype. Protein expression of ABCG2 and P-glycoprotein (P-gp/ABCB1/Mdr-1) in multi-drug resistance model cell lines. Parental cell lines: MDCKII and A2780, cell lines overexpressing indicated ABC transporter: MDCK-II-ABCG2, MDCKII-MDR1 and A2780/Adr (a). Full length blots with indicated position of cropped area presented in the panel a. (b)

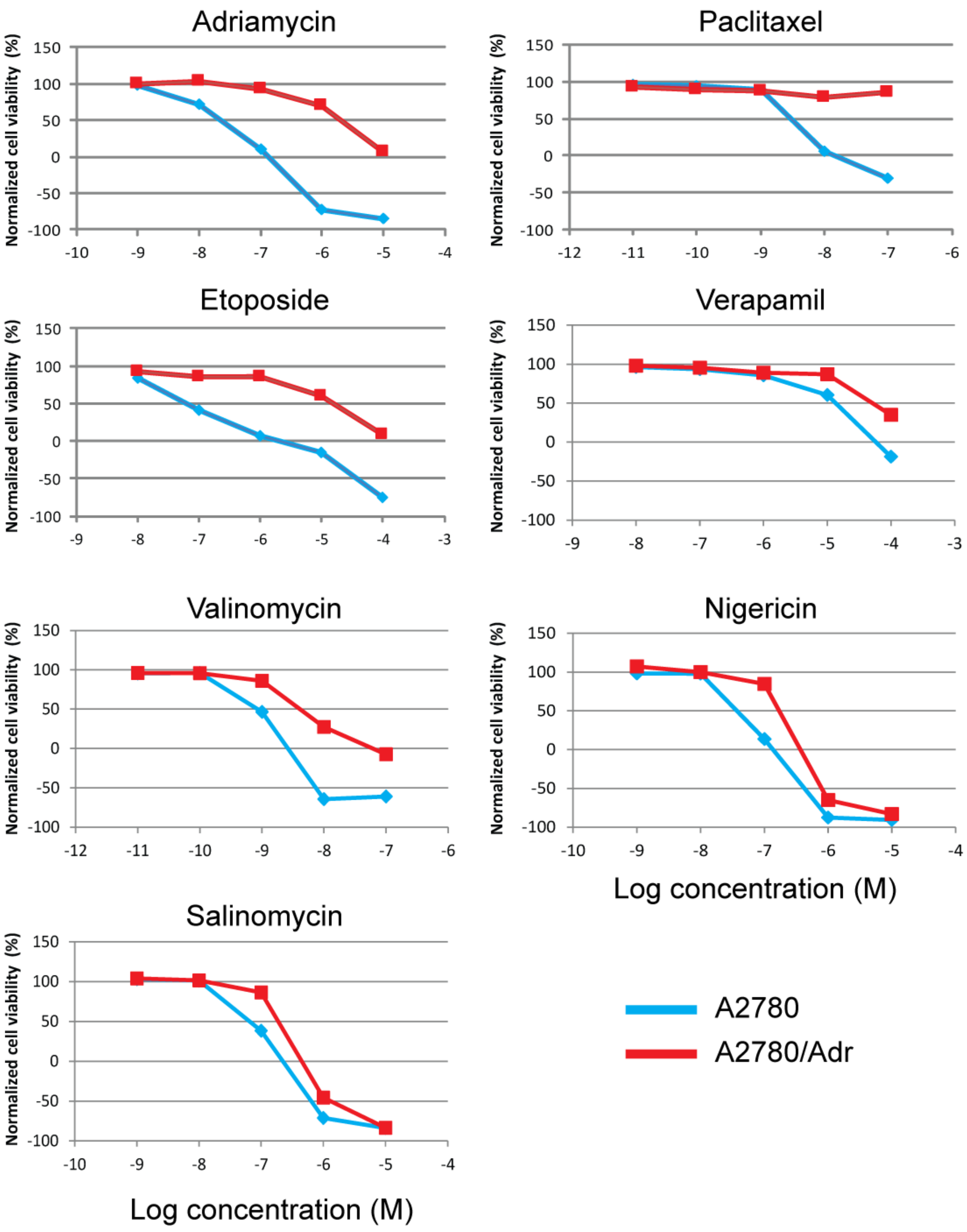

Supplementary Figure S4. **Concentration-response curves of A2780 and A2780/Adr cells.** A2780 and A2780/Adr ovarian carcinoma cells were treated with various chemotherapeutics at concentrations as indicated.

Cell viability inhibition by crown ethers

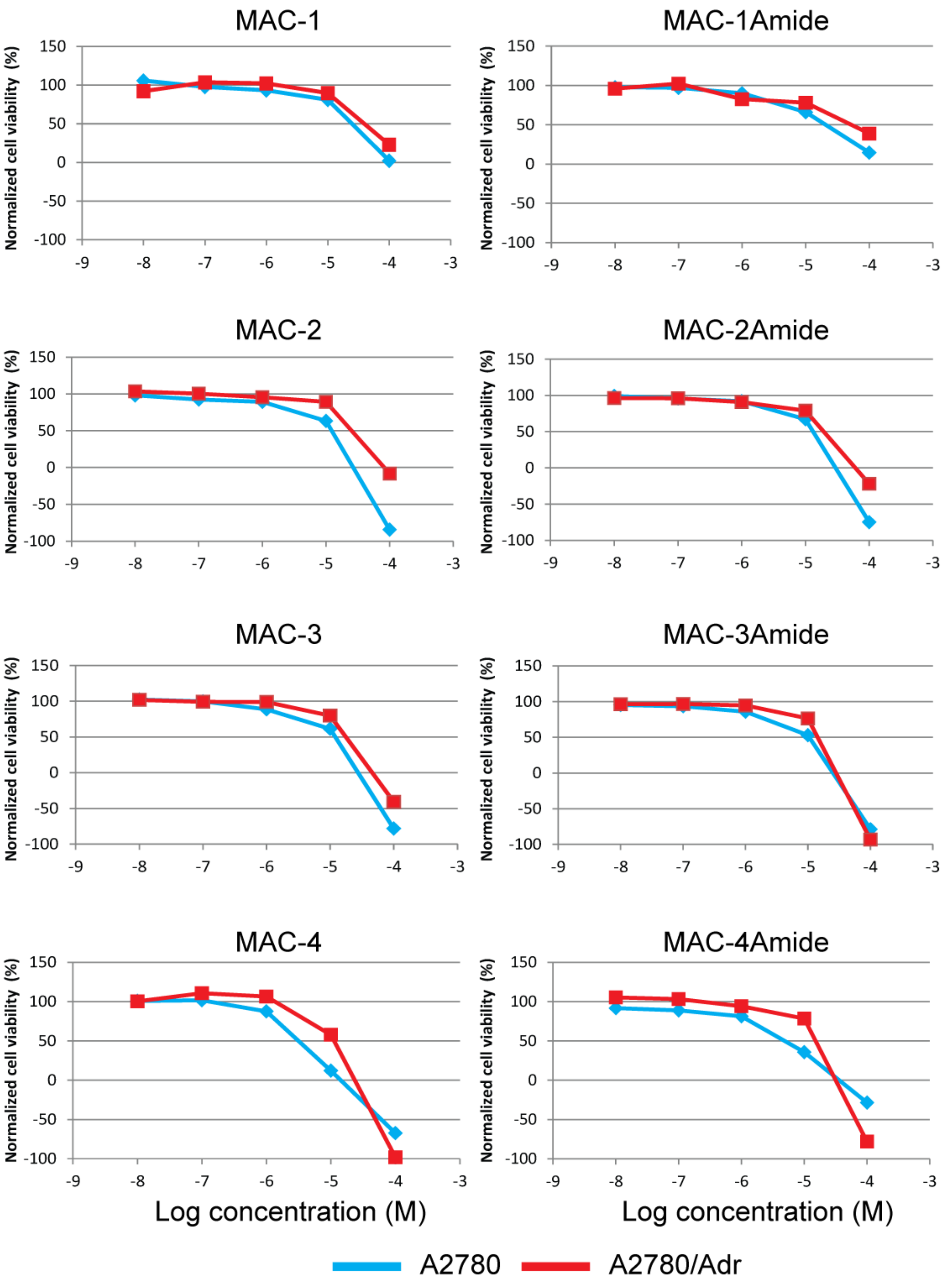

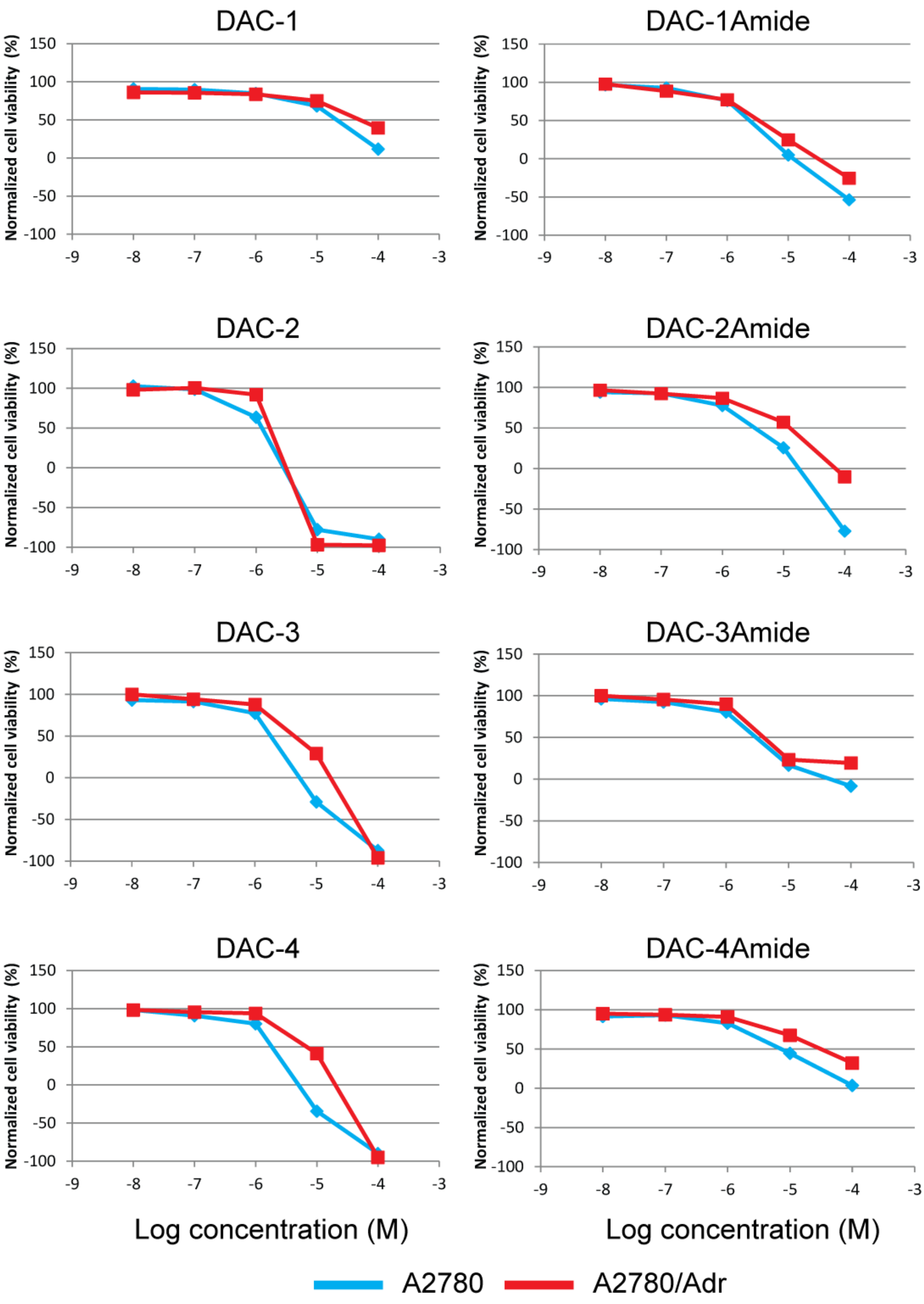

Supplementary Figure S5. **Concentration-response curves of A2780 and A2780/Adr cells.** A2780 and A2780/Adr ovarian carcinoma cells were treated with various crown ethers at concentrations as indicated.

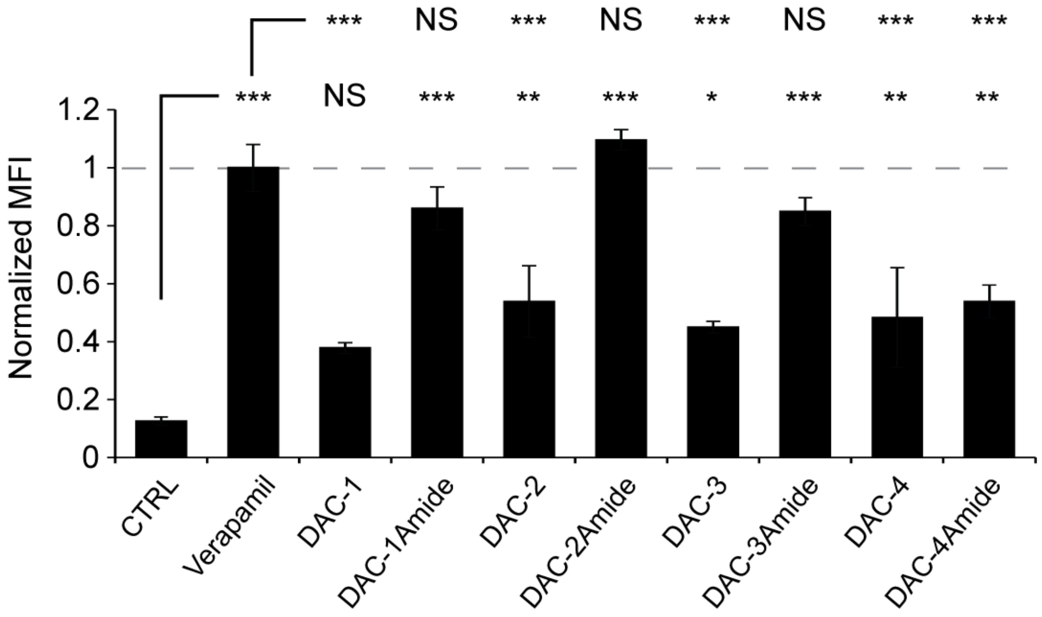

Supplementary Figure S6. P-gp drug efflux is affected by crown ethers. Functional activity of P-gp was evaluated by calcein accumulation assay. Normalized Mean fluorescence intensity (MFI) of calcein in A2780/Adr cells after incubation with 0.25 μM calcein-AM (CTRL) and treatment with verapamil and diaza-crown ethers at 10 μM concentration. All data are shown as mean ± s.d. of 3 individual experiments and normalized to verapamil. One-way ANOVA with Tukey's post-hoc test was used for statistical analysis (NS – non-significant; * - $p < 0.05$, ** - $p < 0.01$, *** - $p < 0.001$).

Sensitization of P-gp overexpressing cells towards paclitaxel and doxorubicin

Supplementary table S3. Crown ethers sensitise resistant A2780/Adr cells to paclitaxel and adriamycin. IC_{50} values (mean ± s.d.) of sensitization experiments were calculated from corresponding cell viability curves plotted from n = 1 – 5 experiments performed in quadruplicates. Abbreviated data from inhibition curves presented as bars corresponding to this table can be viewed in Figure 3A-C and Supplementary Figures S4, S8 and S9. All concentrations are presented in nM (n.t. – not tested).

**Treatment**		**Paclitaxel + compound**		**Adriamycin + compound**	
	**Cell line**	**A2780**	**A2780/Adr**	**A2780**	**A2780/Adr**
**Compound**	**Conc.**				
CTRL		2.9 ± 0.8	> 100	23.2 ± 12.9	2280 ± 1670
Verapamil	10000	1.4	29 ± 6	< 10	21
DAC-2Amide	100	3.4 ± 3.4	> 100	< 10	620 ± 150
	500	3.2 ± 3.5	> 100	< 10	39 ± 0.4
	1000	1.9 ± 0.2	44 ± 13	< 10	27 ± 4.4
	1500	0.4 ± 0.7	33 ± 12	< 10	25 ± 2.2
	2000	< 0.01	34 ± 3	< 10	22 ± 3.5
DAC-3Amide	100	2.8 ± 0.4	> 100	< 10	280 ± 40
	500	2.7 ± 0.5	> 100	< 10	150 ± 40
	1000	1.8 ± 0.5	62 ± 22	< 10	68 ± 3
	1500	0.4 ± 0.9	43 ± 10	< 10	43 ± 7
	2000	1.3 ± 0.3	33 ± 5	< 10	31 ± 8
DAC-4Amide	100	2.4	> 100	n.t.	n.t.
	500	2.5	> 100		
	1000	2.1	> 100		
	1500	0.1	> 100		
	2000	0.1	≥ 100		

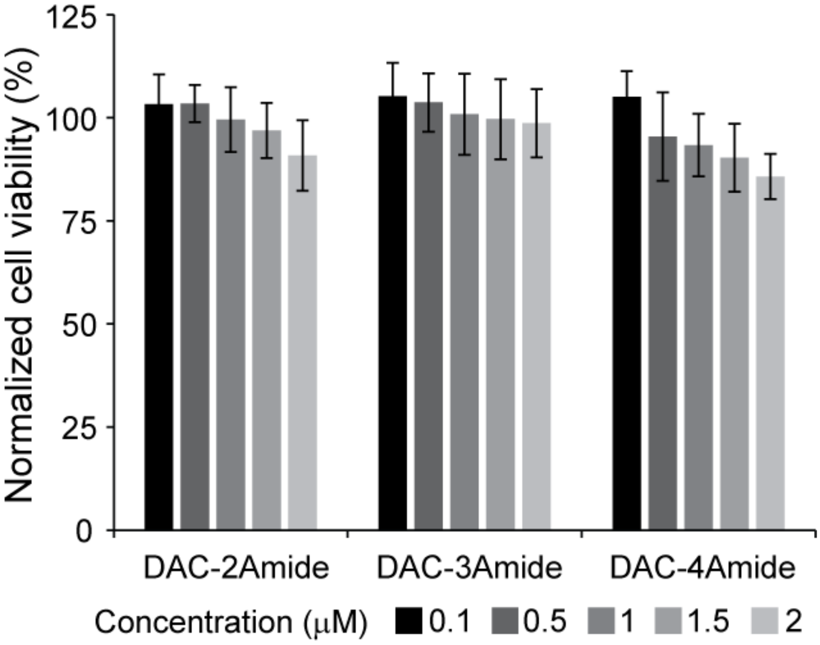

Supplementary Figure S7. Crown ethers do not inhibit growth of resistant cells. A2780/Adr cell line was treated with increasing concentrations of DAC-2Amide, -3Amide or -4Amide. Cell viability was evaluated by MTT assay after 72h of incubation, and the percentage of viable cells was calculated. Each bar represents a mean ± SD of three individual experiments performed in quadruplicates.

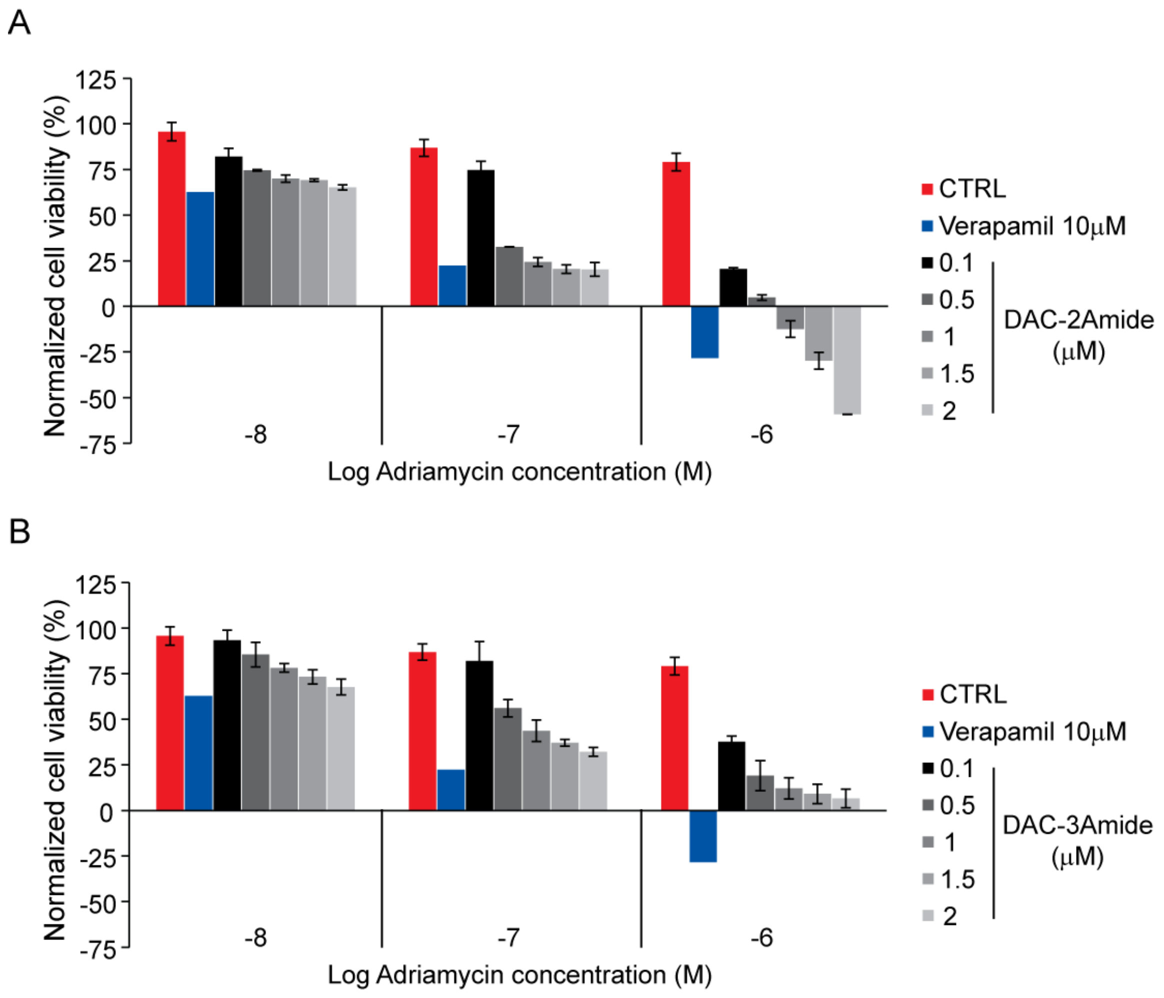

Supplementary Figure S8. Crown-ethers sensitize resistant cells to Adriamycin. A2780/Adr cell line was treated with increasing concentrations of Adriamycin alone (CTRL) or in combination with verapamil or tested crown ethers as indicated. Cell viability was evaluated by MTT assay after 72h of incubation, and the percentage of viable cells was calculated. Each bar represents a mean value ± SD of at least three individual experiments performed in quadruplicates except for verapamil where n = 1.

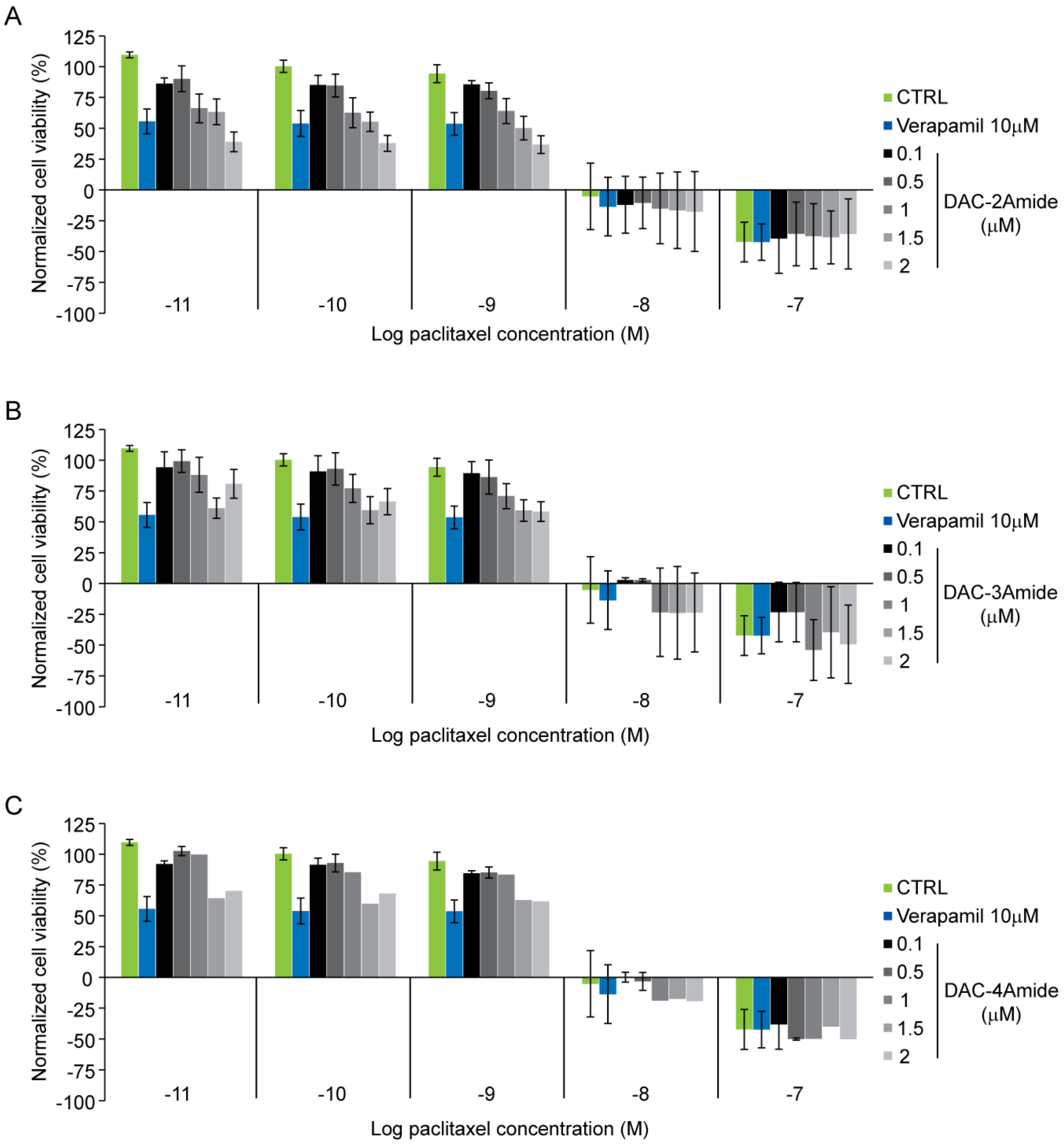

Supplementary Figure S9. Crown ethers do not sensitize non-resistant cells to paclitaxel. A2780 cell line was treated with increasing concentrations of paclitaxel alone (CTRL) or in combination with verapamil or tested crown ethers as indicated. Cell viability was evaluated by MTT assay after 72h of incubation, and the percentage of viable cells was calculated. Each bar represents a mean value ± SD of at least three individual experiments performed in quadruplicates except for DAC-4Amide where n = 1.

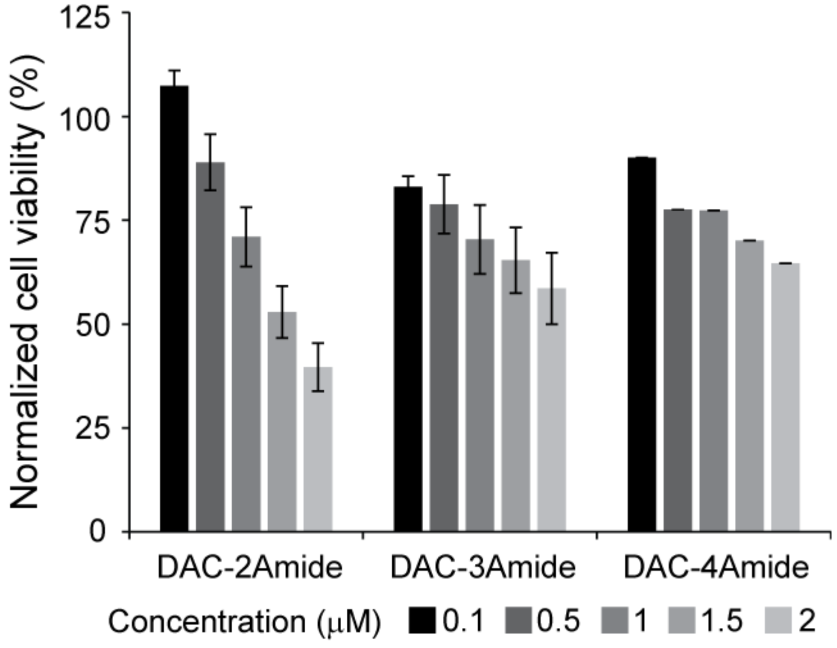

Supplementary Figure S10. Crown ethers inhibit growth of non-resistant cells. A2780 cell line was treated with increasing concentrations of DAC-2Amide, -3Amide or -4Amide. Cell viability was evaluated by MTT assay after 72h of incubation, and the percentage of viable cells was calculated. Each bar represents a mean ± SD of at least three individual experiments performed in quadruplicates except for DAC-4Amide where n = 1.

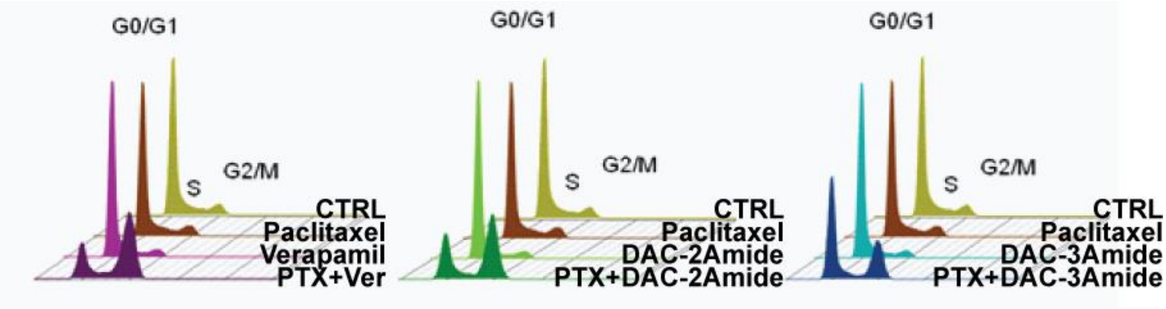

Supplementary Figure S11. Cell cycle analysis. Representative flow cytometry histograms of DNA content analysis. A2780/Adr cells were cultured without treatment (CTRL), treated with compounds alone as indicated (0.1 µM paclitaxel, PTX; 10 µM verapamil, Ver; 1 µM DAC-2Amide and -3Amide), or in combination with paclitaxel for 48h.

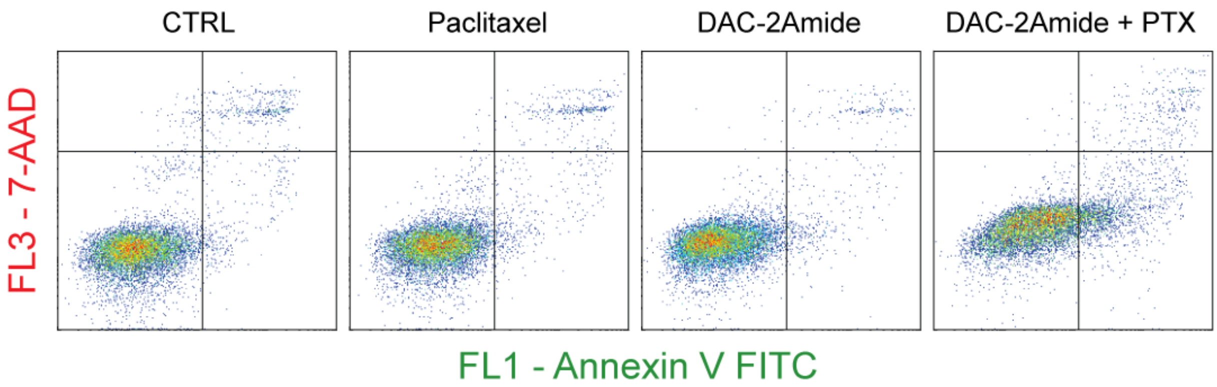

Supplementary Figure S12. Annexin V assay. Representative Annexin V / 7-AAD flow cytometry dot plots (DAC-2Amide data only) A2780/Adr cells were cultured without treatment (CTRL), treated with compound alone as indicated (0.1 µM paclitaxel, PTX; 1 µM DAC-2Amide), or in combination for 48h.

Effects of long-term exposure of crown ethers on P-gp expression and functionality

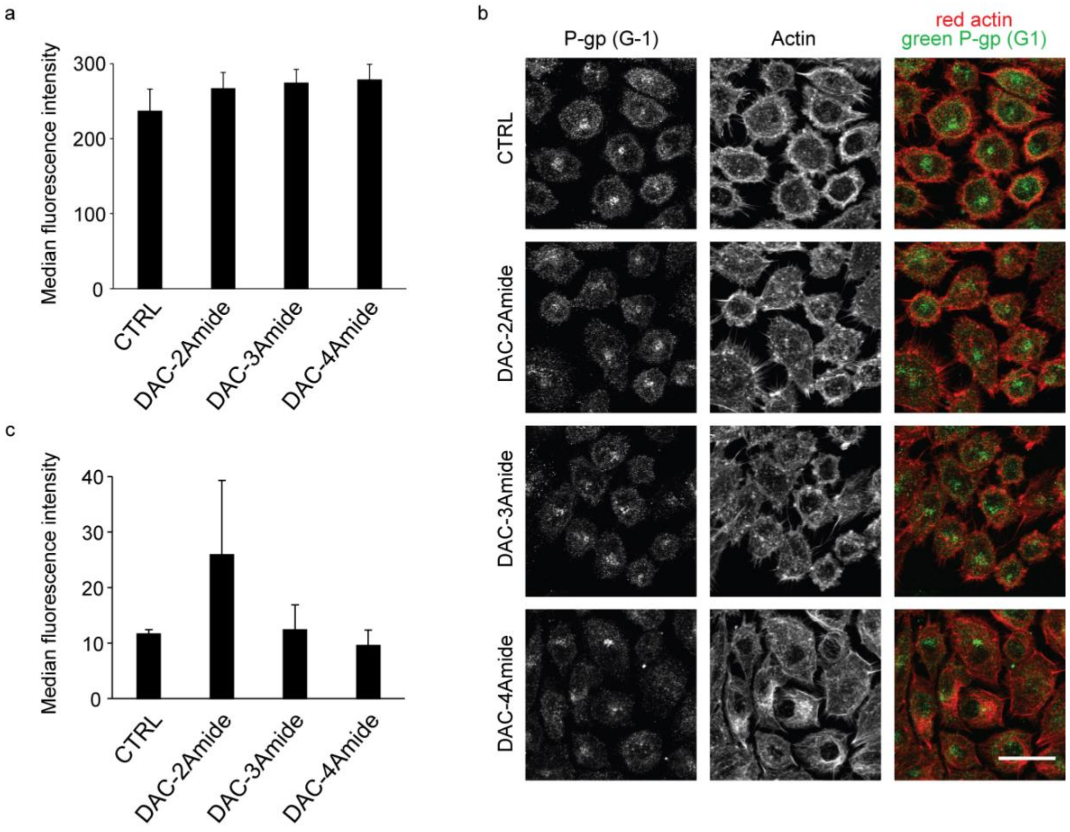

Supplementary Figure S13. Effects of long-term exposure of crown ethers on P-gp expression and functionality. Median fluorescence intensity of P-gp expression at the plasma-membrane analyzed by flow cytometry using anti-P-gp antibody that recognizes extracellular epitope of the transporter (clone 4E3) (**A**). A2780/Adr cells were cultured without treatment (CTRL) or treated with compounds DAC-2Amide, -3Amide or -4Amide (2 μM) for 72h. Immunofluorescent staining with phalloidin (actin, red) and anti-P-gp antibody that recognises intracellular epitope (clone G1, green). Maximum projection of 10 optical slices is shown (scale bar = 20 μm) (**B**). Median fluorescence intensity of Rhodamine 123 in A2780/Adr cells performed after long-term exposure to crown-ethers. Rho efflux was performed without addition of compounds (**C**). Each bar represents a mean value ± s.d. from n = 4 (A) and n = 3 (C) individual experiments. One-way ANOVA with Dunnett's post-hoc test was used for statistical analysis (all treated samples were not significant as compared to control)

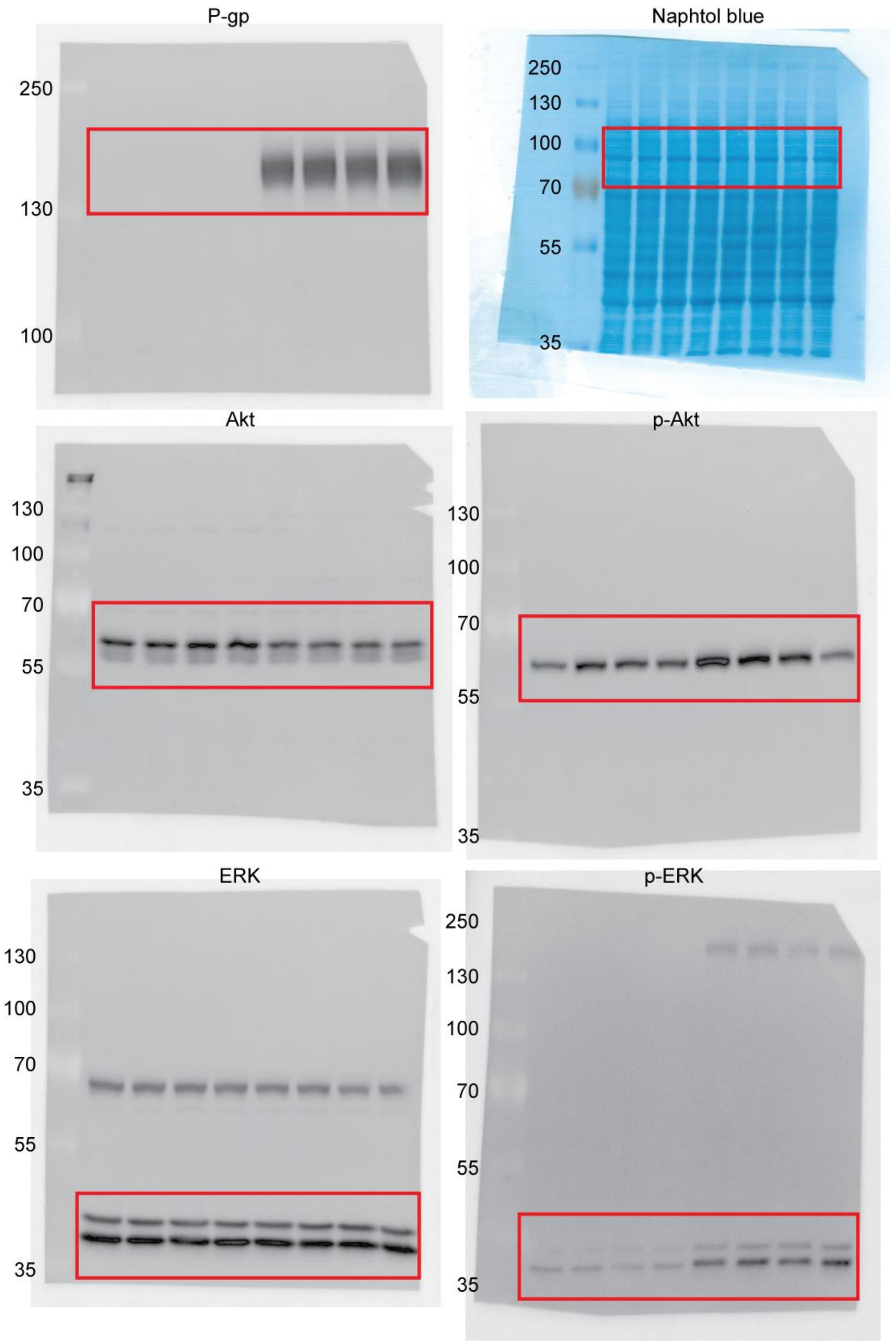

Supplementary Figure S14. Full length blots corresponding to Figure 5. in the main text with indicated cropped area. ERK antibody detects an unspecific band at ~69 kDa. Faint band at ~170 kDa in p-ERK blot correspond to previous staining of the same membrane with P-gp antibody.

References

1. Schultz, R. A., White, B. D., Dishong, D. M., Arnold, K. A. & Gokel, G. W. 12-membered, 15-membered, and 18-membered-ring nitrogen-pivot lariat ethers-syntheses, properties, and sodium and ammonium cation binding properties**.** *J. Am. Chem. Soc.* **107,** 6659-6668 (1985).

2. Gatto, V. J. *et al*. Syntheses and binding-properties of bibrachial lariat ethers (bibles)-survey of synthetic methods and cation selectivities. *J. Org. Chem.* **51,** 5373–5384 (1986).

3. K. Mlinarić-Majerski, T. Šumanovac Ramljak*, Tetrahedron*. **58** (2002) 4893-4898.

4. Kralj, M., Majerski, K., Sumanovac Ramljak, T. & Marjanovic, M. Adamantane derivatives of AZA-crown ethers and their use in treatment of tumor. US 8389505 B2

5. Supek, F. *et al*. Could LogP be a principal determinant of biological activity in 18-crown-6 ethers? Synthesis of biologically active adamantane-substituted diaza-crowns. *Eur. J. Med. Chem.* **46,** 3444-3454 (2011).

6. Šumanovac Ramljak,T., Mlinarić-Majerski, K. & Bertoša, B. Alkali metal ion complexation of adamantane functionalized diaza-bibracchial lariat ethers. *Croat. Chem. Acta* **85**, 559-568 (2012).

7. Henkel, J.G., Hane, J.T. & Gianutsos, G. Structure-antiparkinson activity relationships in the aminoadamantanes. Influence of bridgehead substitution. *J. Med. Chem.* **25,** 51−56 (1982);

8. Stetter, H., Schwartz, M. & Hirschhorn, A. Uber verbindungen mit urotropin-struktur.12. Monofunktionelle adamantan-derivate. *Chem. Ber.-Rec.* **92,** 1629−1635 (1959);

9. Lunn, W., Podmore, W. & Szinai, S. Adamantane chemistry. I. synthesis of 1,2 disubstituted adamantanes. *J. Chem. Soc. C-Org.* **13,** 1657−1660 (1968);

10. Oppolzer, W. & Moretti, R. Enantioselective syntheses of alpha-amino-acids from 10-sulfonamido-isobornyl esters and di-trans-butyl azodicarboxylate. *Tetrahedron* **44,** 5541−5552 (1988);

11. Fieser, L.F., Nazer, M.Z., Archer, S., Berberian, D.A. & Slighter, R.G. Naphthoquinone antimalarials.30.2-hydroxy-3-⌈omega-(1-adamantyl)alkyl⌋-1,4-naphthoquinones. *J. Med. Chem.* **10**, 517-521 (1967).

12. Marchand, A.P., Kumar, K.A., McKim, A.S., Mlinarić-Majerski, K. & Kragol, G. Synthesis and alkali metal picrate extraction capabilities of novel cage functionalized 17-crown-5 and 17-crown-6 ethers. *Tetrahedron* **53,** 3467−3474 (1979);

13. Mlinarić-Majerski, K. & Kragol, G. Design, synthesis and cation-binding properties of novel adamantane- and 2-oxaadamantane-containing crown ethers. *Tetrahedron* **57,** 449−457 (2001);
14. Marchand, A. P., *et al*., Stabilization of a K+-(bis-cage-annulated 20-crown-6) complex by bidentate picrate *Struct. Chem.* **14** 279−288 (2003).
